# Supplementary material for: Genome-wide association analysis reveals new insights into the genetic architecture of defensive, agro-morphological and quality-related traits in cassava
Source: Plant Mol Biol. 2020 Jul 30;109(3):195–213. doi: 10.1007/s11103-020-01038-3 (PMC9162993; doi:10.1007/s11103-020-01038-3)
Supplement: Supplementary file 1 — Supplementary material 1 (PDF 14169.1 kb) [file 11103_2020_1038_MOESM1_ESM.pdf]

Electronic supplementary materials

Title: Cassava GWAS catalogue reveals new insights into the genetic architecture of defensive, morphological and quality-related traits

Journal: Plant Molecular Biology

Author names: Ismail Yusuf Rabbi[1*], Siraj Ismail Kayondo[1], Guillaume Bauchet[2], Muyideen Yusuf[1], Cynthia Aghogho[1], Kayode Ogunpaimo[1], Ruth Uwugiaren[1], Ikpan Andrew Smith[1], Prasad Peteti[1], Afolabi Agbona[1], Elizabeth Parkes[1], Ezenwaka Lydia[3], Marnin Wolfe[5], Jean-Luc Jannink[5,6], Chiedozie Egesi[1,3,4], and Peter Kulakow[1]

Affiliations

[1] International Institute of Tropical Agriculture (IITA), Ibadan, Oyo State, 200001 Nigeria,
[2] Boyce Thompson Institute, Ithaca, New York 14853;
[3] National Root Crops Research Institute (NRCRI), PMB 7006, Umudike, 440221 Nigeria;
[4] Global Development Department, College of Agriculture and Life Sciences, Cornell Univ., Ithaca, New York 14850;
[5] Section on Plant Breeding and Genetics, School of Integrative Plant Sciences, Cornell Univ., Ithaca, NY 14850;
[6] United States Department of Agriculture - Agriculture Research Service, Ithaca, New York 14850;

* Corresponding author: I.Rabbi@cgiar.org

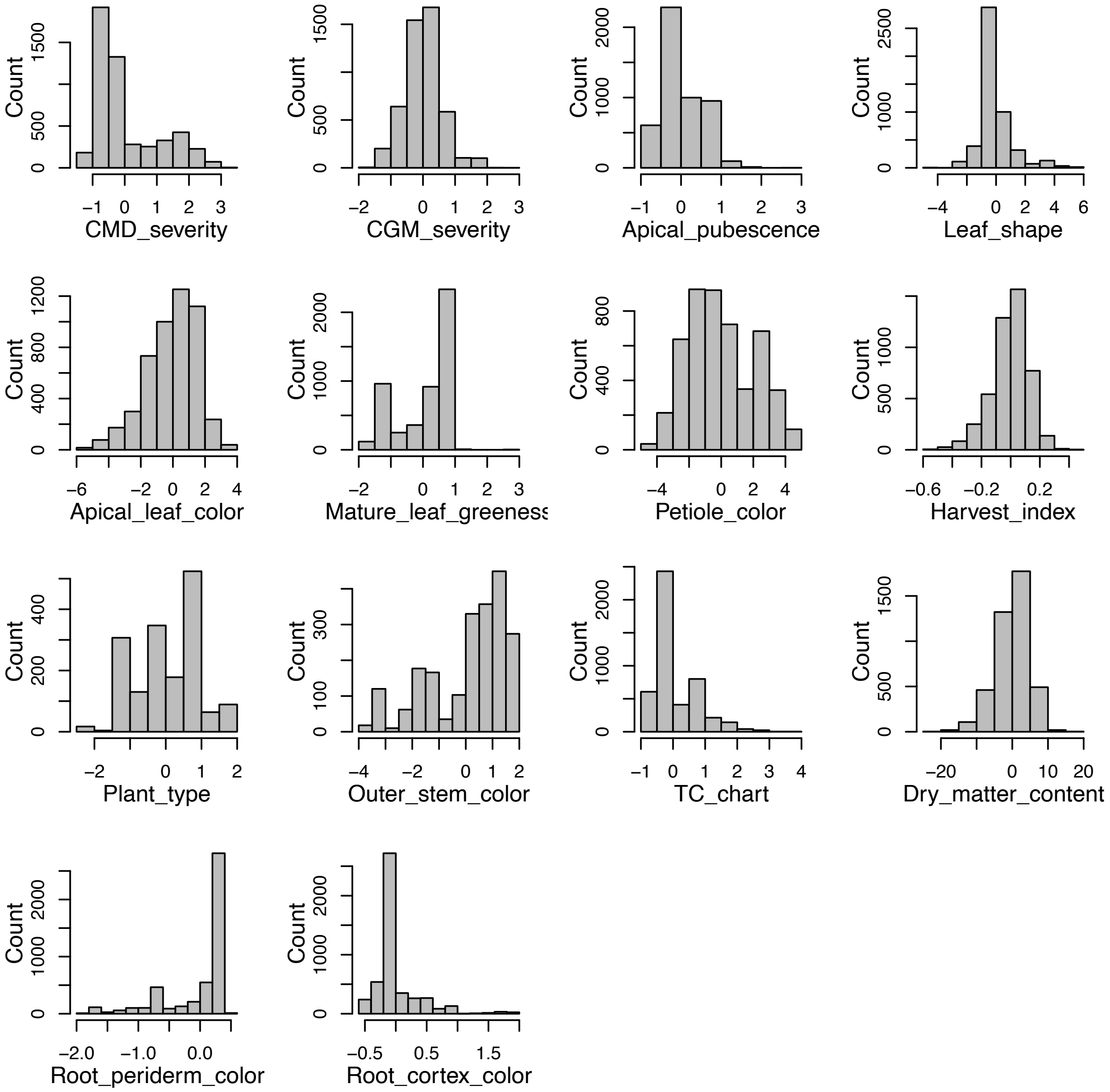

Figure S1 Histogram showing the distribution of BLUPs for the 14 traits used in the GWAS analysis.

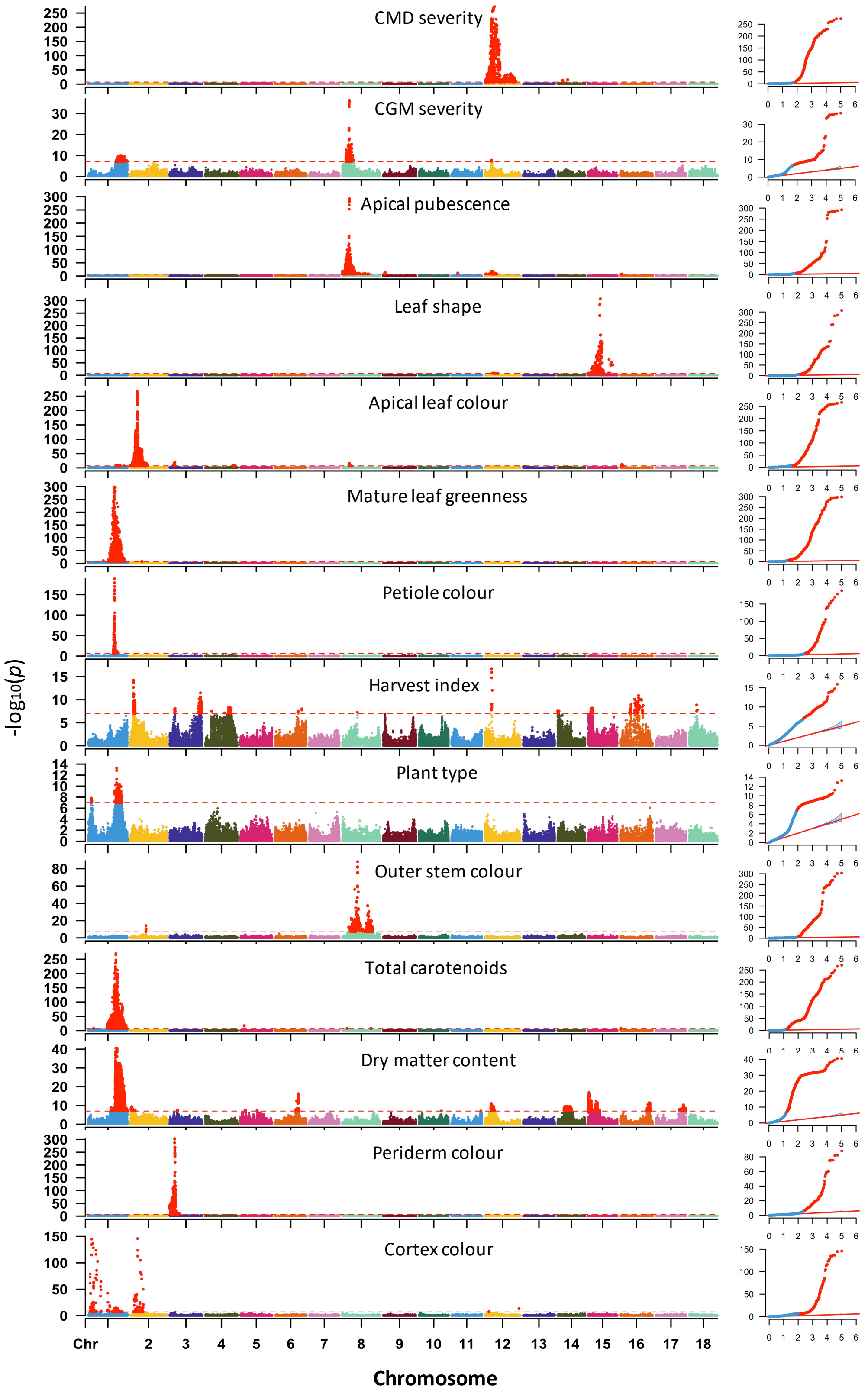

Figure S2 Manhattan plots for GWAS for 14 traits of 5,130 cassava accessions using "MLMe" analysis approach. A total of 101,521 SNP markers were used for that GWAS analyses with the red horizontal line representing genome-wide significance threshold (α=0.05/101521 = 4.93 × 10^{-7}). The QQ-plots inset - right with observed p-values on the y-axis and expected p-values on the x-axis.

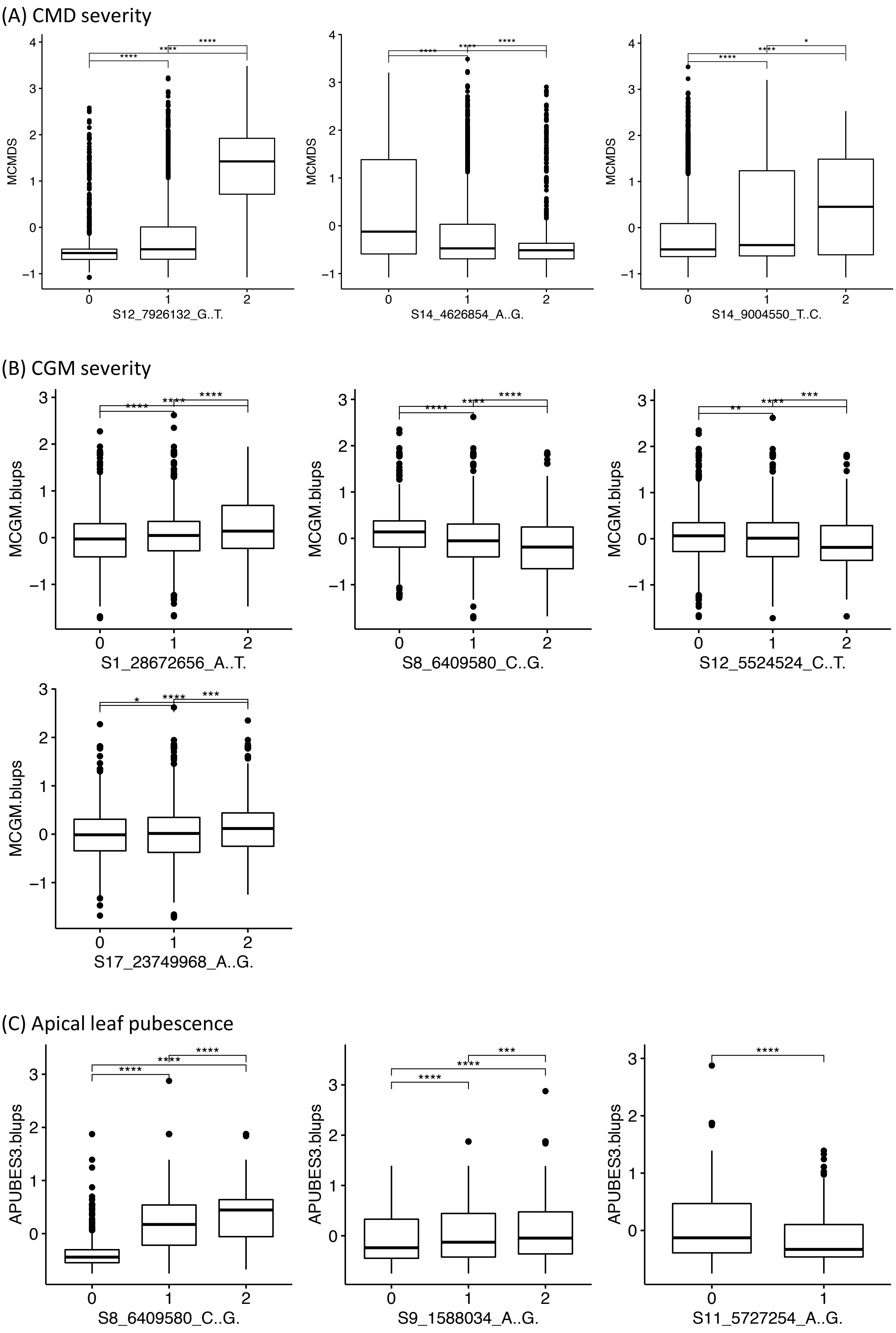
(A) CMD severity
MCMDS
S12_7926132_G..T.
S14_4626854_A..G.
S14_9004550_T..C.
(B) CGM severity
MCGM.blups
S1_28672656_A..T.
S8_6409580_C..G.
S12_5524524_C..T.
S17_23749968_A..G.
(C) Apical leaf pubescence
APUBES3.blups
S8_6409580_C..G.
S9_1588034_A..G.
S11_5727254_A..G.

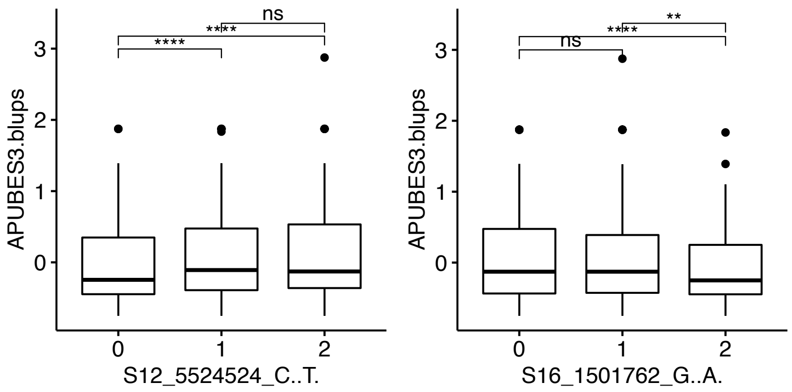

(D) Leaf shape

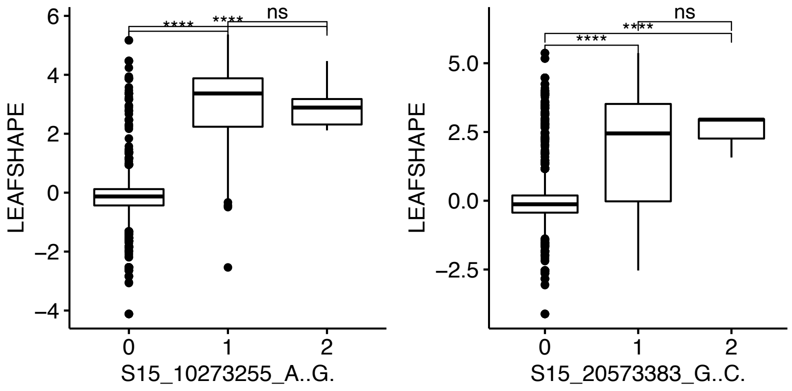

(E) Apical leaf colour

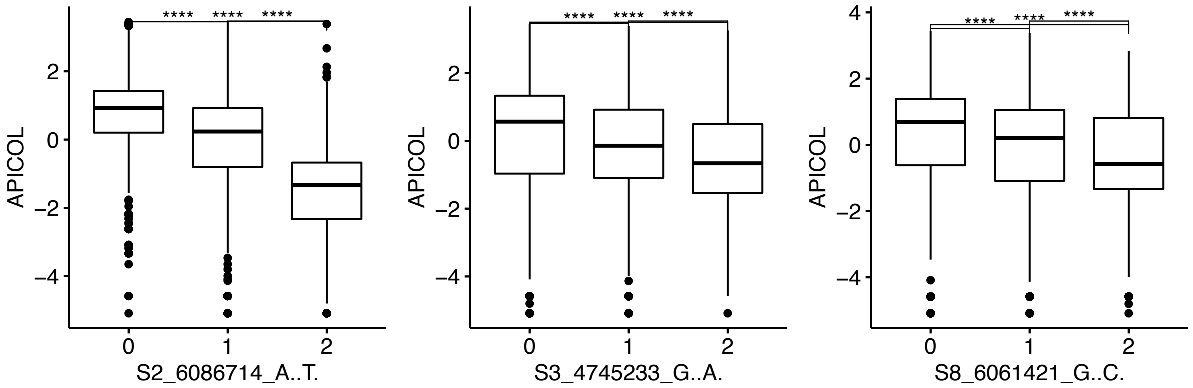

(F) Mature leaf greenness

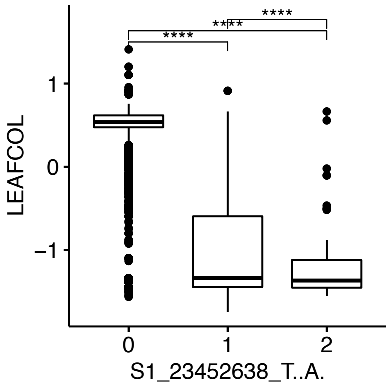

(G) Petiole colour

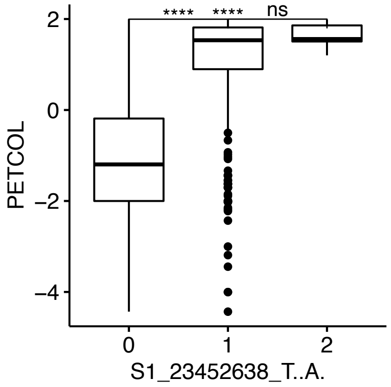

(H) Harvest index

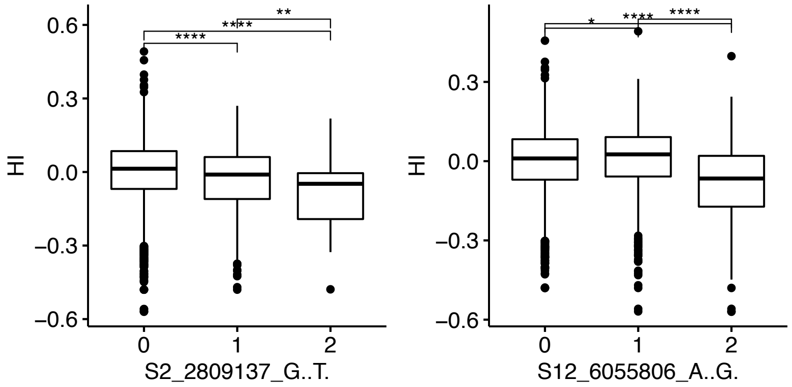

(I) Plant type

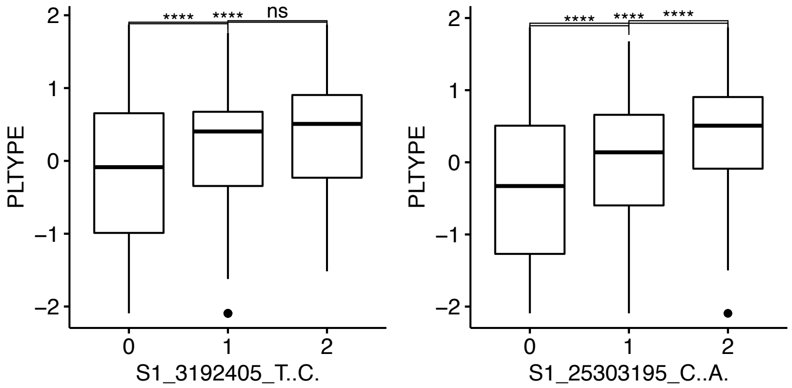

(J) Outer stem colour

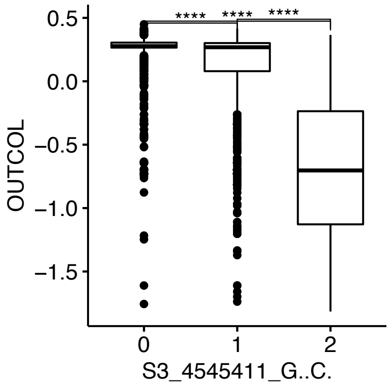

(K) Total carotenoids content

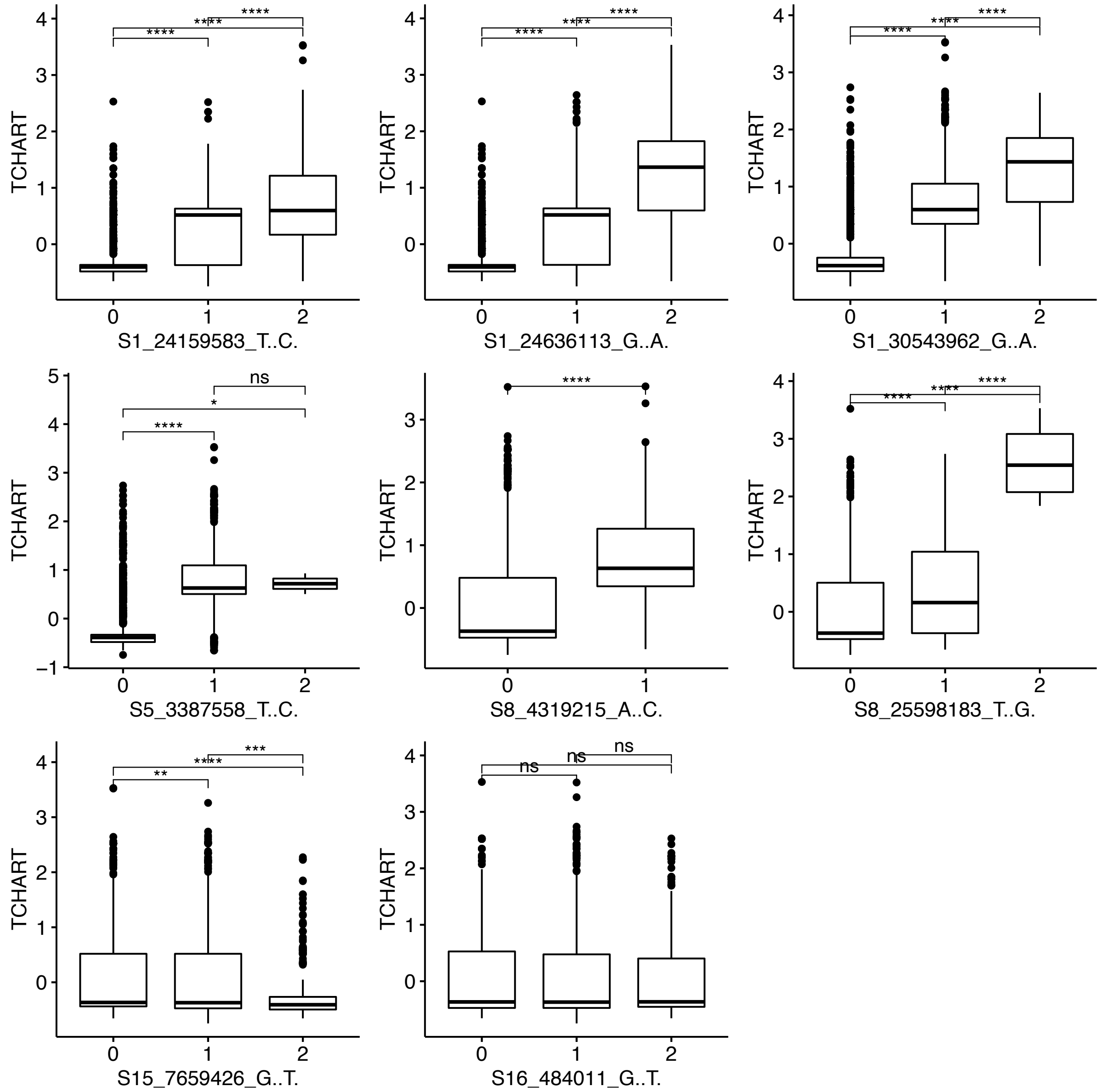

(L) Dry matter content

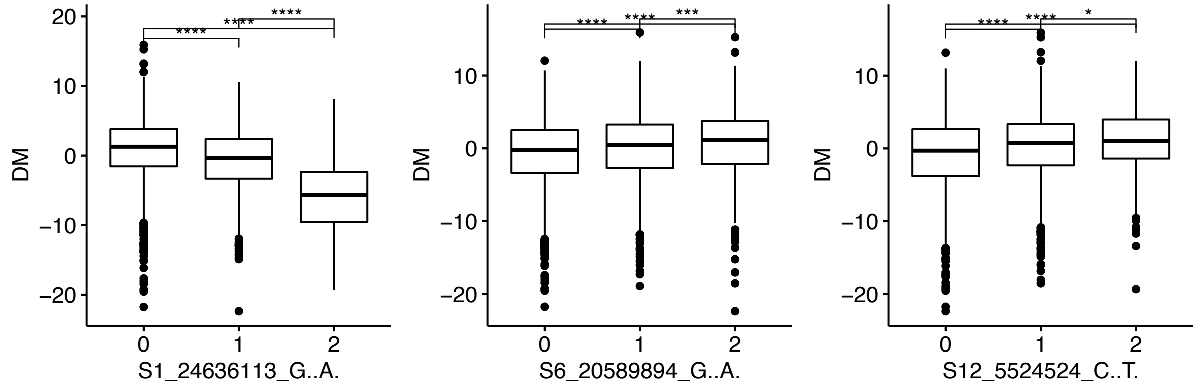

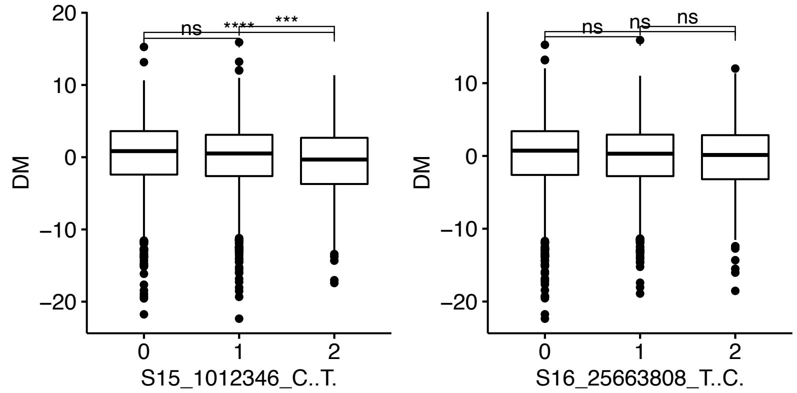

(M) Periderm colour

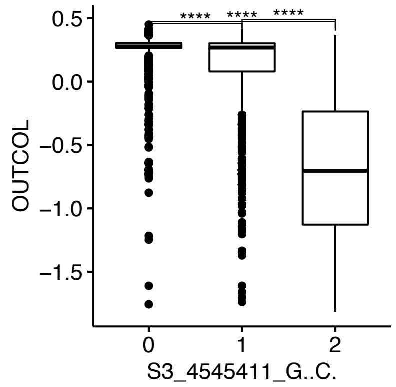

(N) Root cortex colour

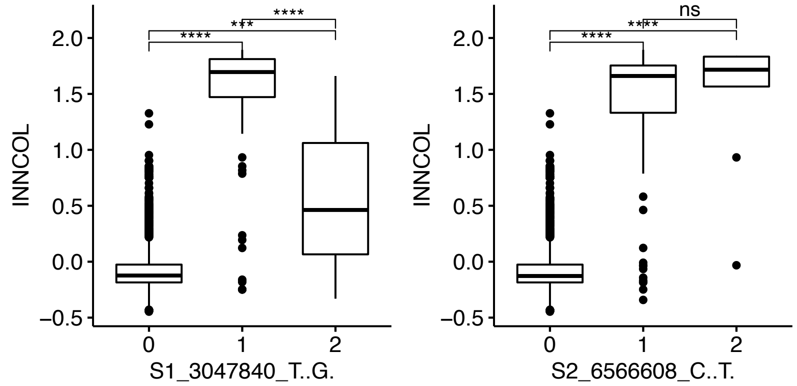

Figure S3 Allelic substitution effects in the most significant SNP at each locus identified for each of the 14 studied traits. Trait BLUPs distribution on y-axis and SNP genotype status of the marker on x-axis. SNPs were converted to dosage format, where 0, 1, and 2 indicates the copies of the minor alleles. The first allele in the suffix of a SNP name denotes the allele being counted in the dosage coding. For example, dosage score of 2 in SNP S12_7926132_G..T. means homozygous for “G”, a score of 1 means heterozygote “GT”, and a score of 0 means homozygote alternate allele “T”. (A) CMD severity; (B) CGM severity; (C) Apical leaf pubescence; (D) Leaf shape(E); Apical leaf colour; (F) Mature leaf greenness; (G) Petiole colour; (H) Harvest index; (I) Plant type; (J) Outer stem colour; (K) Total carotenoids content; (L) Dry matter content; (M) Periderm colour; (N) Root cortex colour. Significance of pairwise t-test between the genotypic classes at each SNP presented. ns: $p > 0.05$; *: $p \leq 0.05$; **: $p \leq 0.01$; ***: $p \leq 0.001$; ****: $p \leq 0.0001$.

A

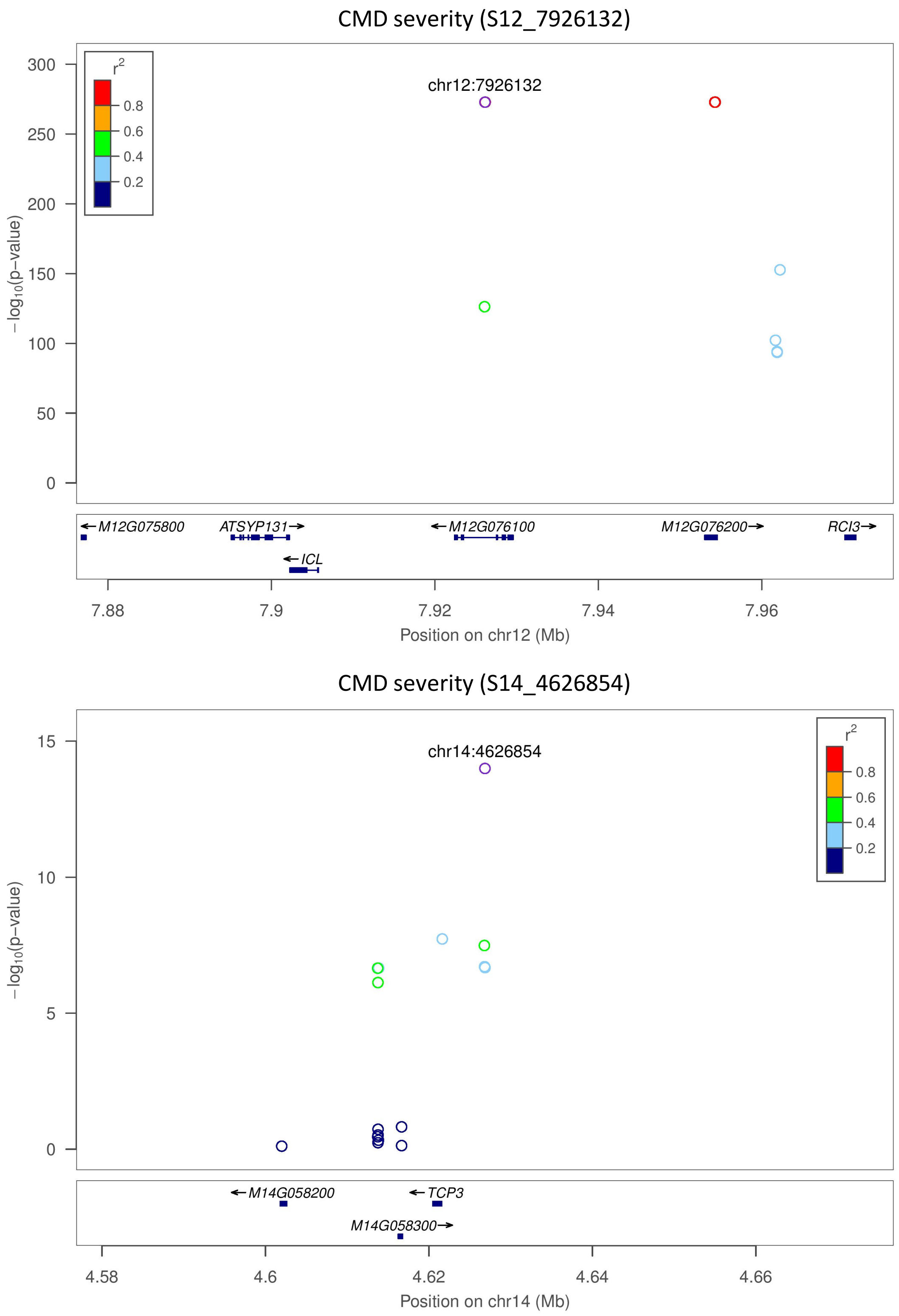
CMD severity (S12_7926132)
chr12:7926132
r2
0.8
0.6
0.4
0.2
300
250
200
150
100
50
0
−log10(p−value)
M12G075800
ATSYP131
ICL
M12G076100
M12G076200
RCI3
7.88
7.9
7.92
7.94
7.96
Position on chr12 (Mb)
CMD severity (S14_4626854)
chr14:4626854
15
10
5
0
M14G058200
TCP3
M14G058300
4.58
4.6
4.62
4.64
4.66
Position on chr14 (Mb)

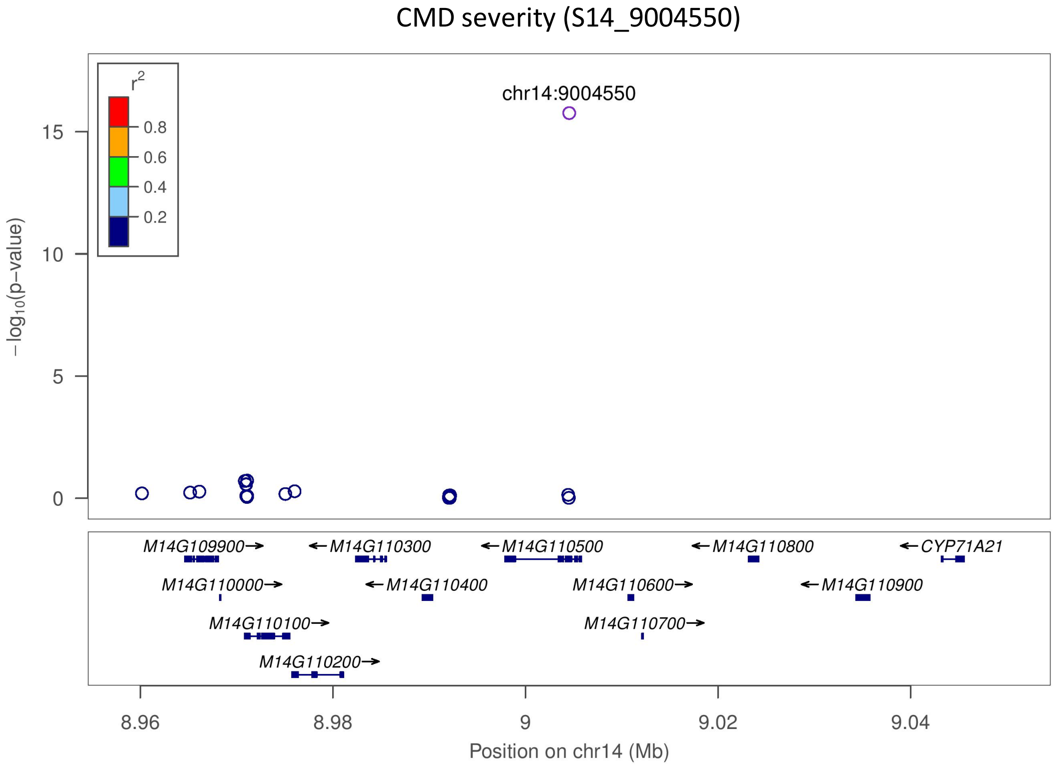
CMD severity (S14_9004550)
chr14:9004550
r2
0.8
0.6
0.4
0.2
−log10(p−value)
0
5
10
15
M14G109900→
←M14G110300
←M14G110500
←M14G110800
←CYP71A21
M14G110000→
←M14G110400
M14G110600→
←M14G110900
M14G110100→
M14G110700→
M14G110200→
8.96
8.98
9
9.02
9.04
Position on chr14 (Mb)

B

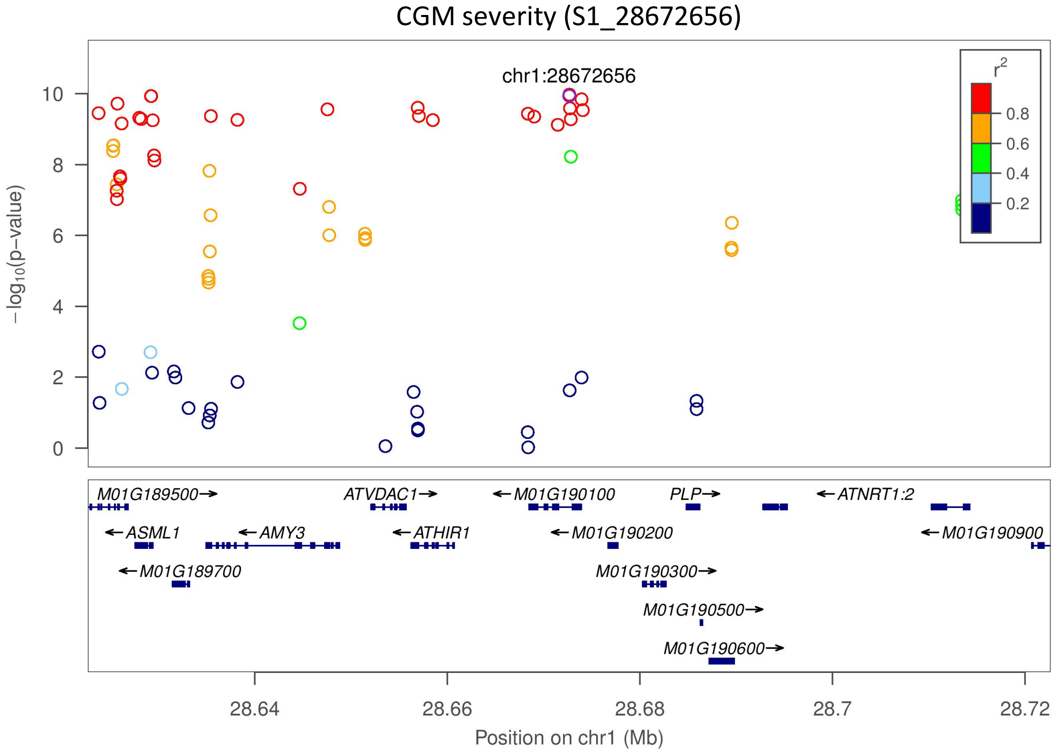
CGM severity (S1_28672656)
chr1:28672656
r2
0.8
0.6
0.4
0.2
−log10(p−value)
0
2
4
6
8
10
M01G189500→
ATVDAC1→
←M01G190100
PLP→
←ATNRT1:2
←ASML1
←AMY3
←ATHIR1
←M01G190200
←M01G190900
←M01G189700
M01G190300→
M01G190500→
M01G190600→
28.64
28.66
28.68
28.7
28.72
Position on chr1 (Mb)

CGM severity (S8_6409580)

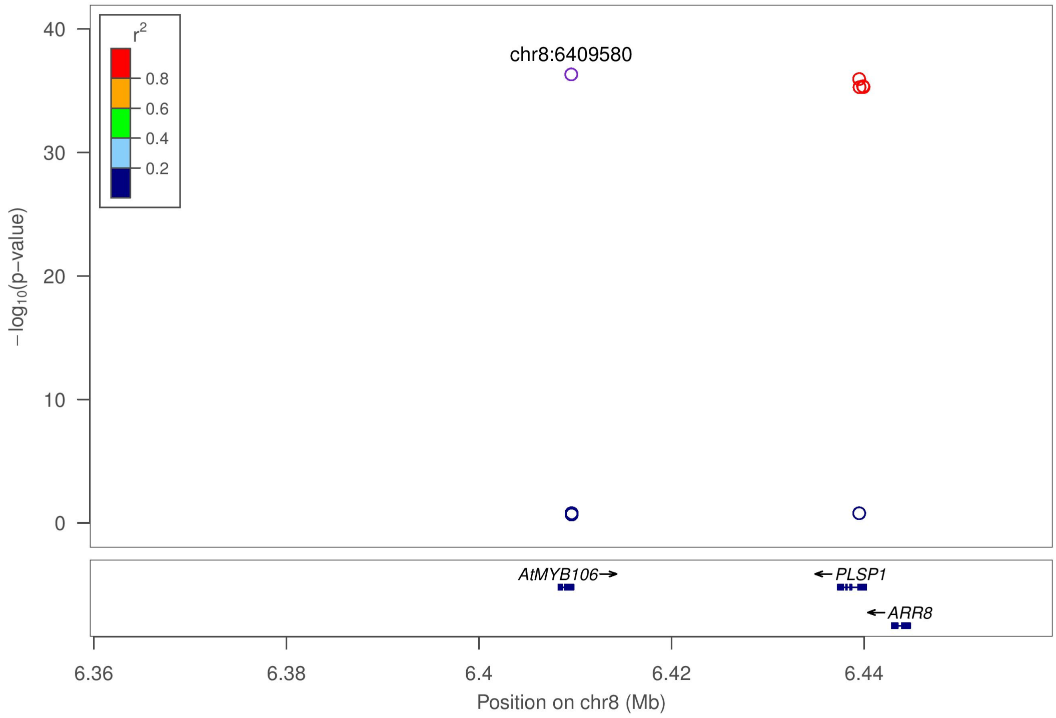

CGM severity (S12_5524524)

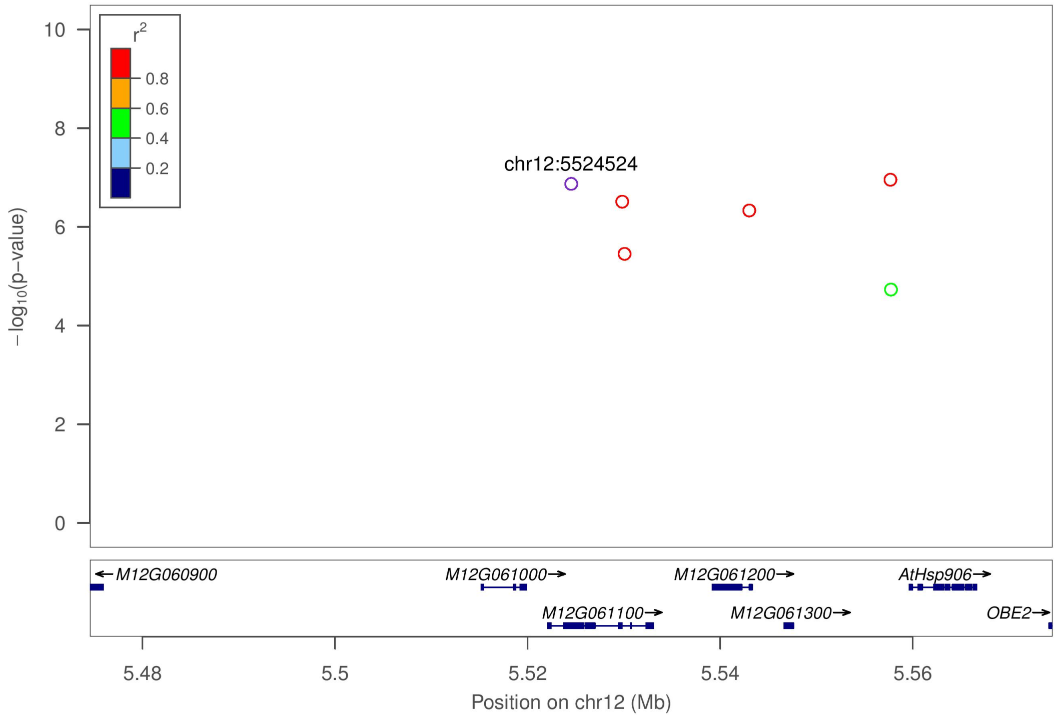

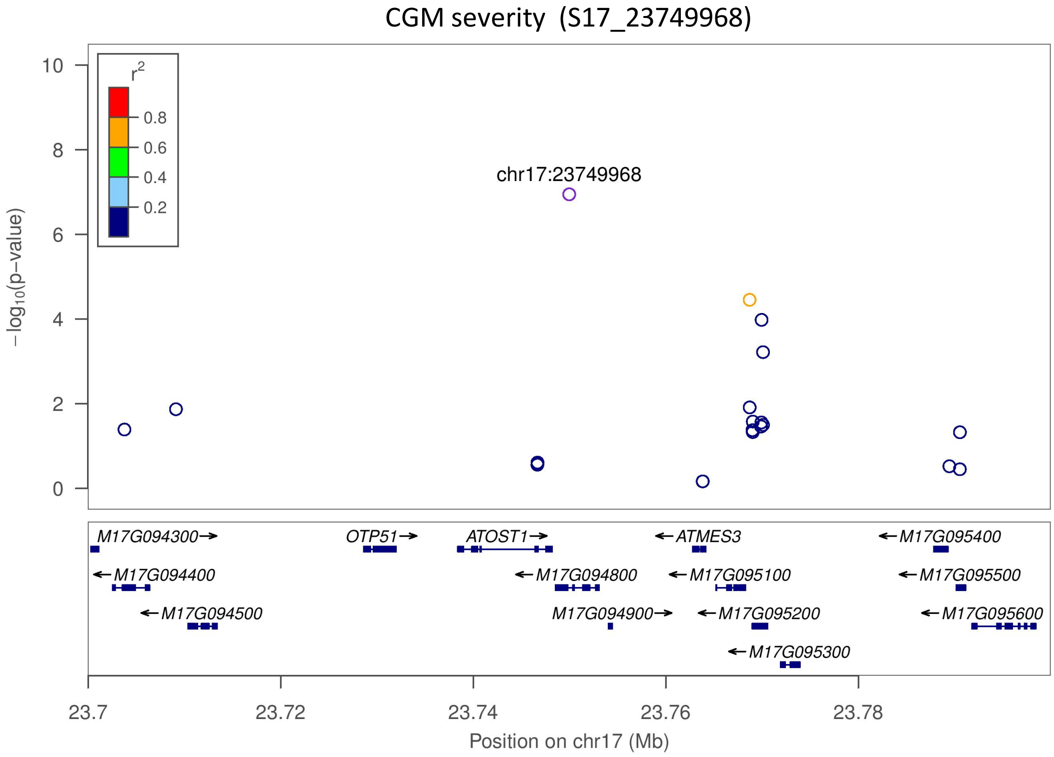
CGM severity (S17_23749968)
chr17:23749968
r2
0.8
0.6
0.4
0.2
10
8
6
4
2
0
−log10(p−value)
M17G094300
OTP51
ATOST1
ATMES3
M17G095400
M17G094400
M17G094800
M17G095100
M17G095500
M17G094500
M17G094900
M17G095200
M17G095600
M17G095300
23.7
23.72
23.74
23.76
23.78
Position on chr17 (Mb)

C

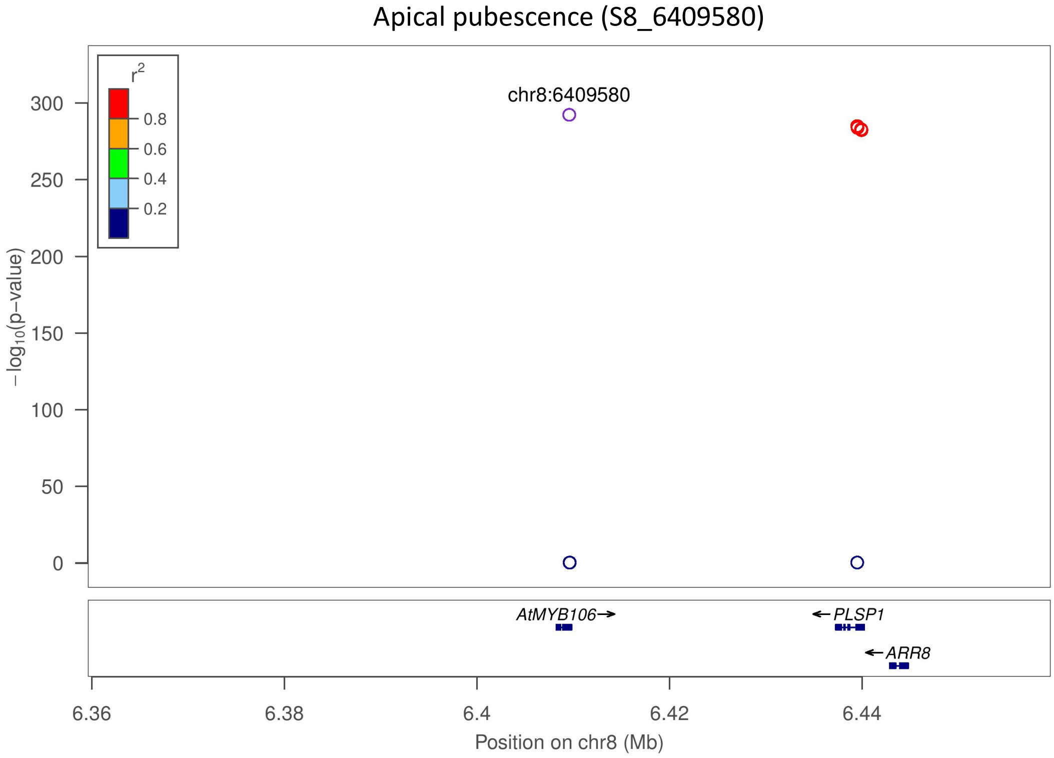
Apical pubescence (S8_6409580)
chr8:6409580
r2
0.8
0.6
0.4
0.2
300
250
200
150
100
50
0
−log10(p−value)
AtMYB106
PLSP1
ARR8
6.36
6.38
6.4
6.42
6.44
Position on chr8 (Mb)

Apical pubescence (S9_1588034)

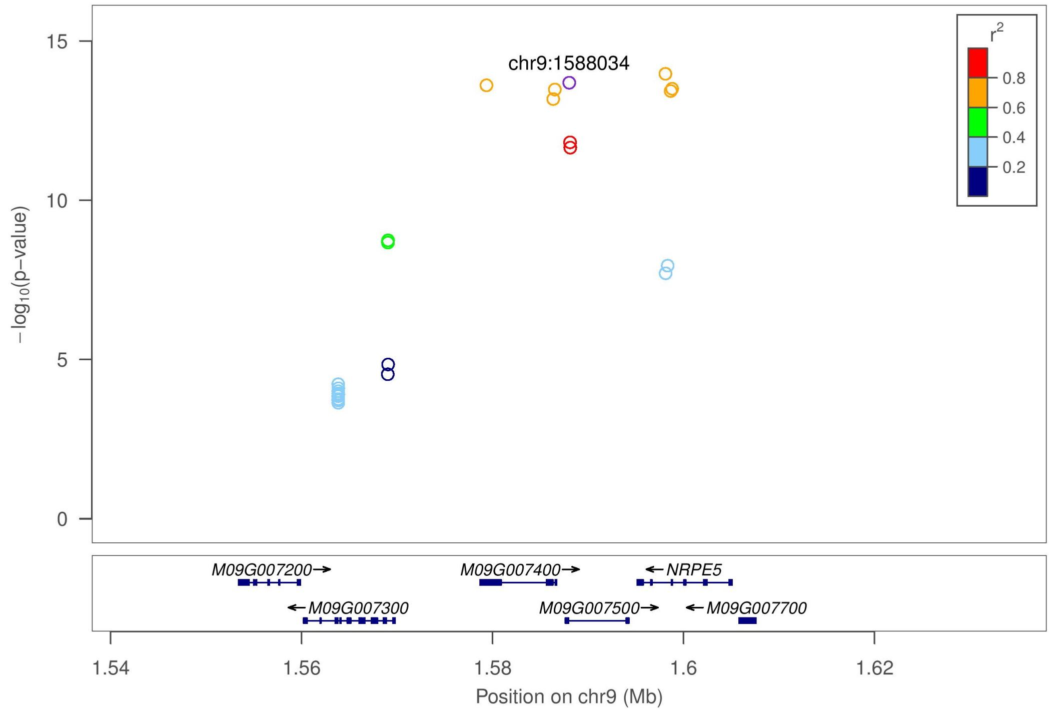

Apical pubescence (S11_5727254)

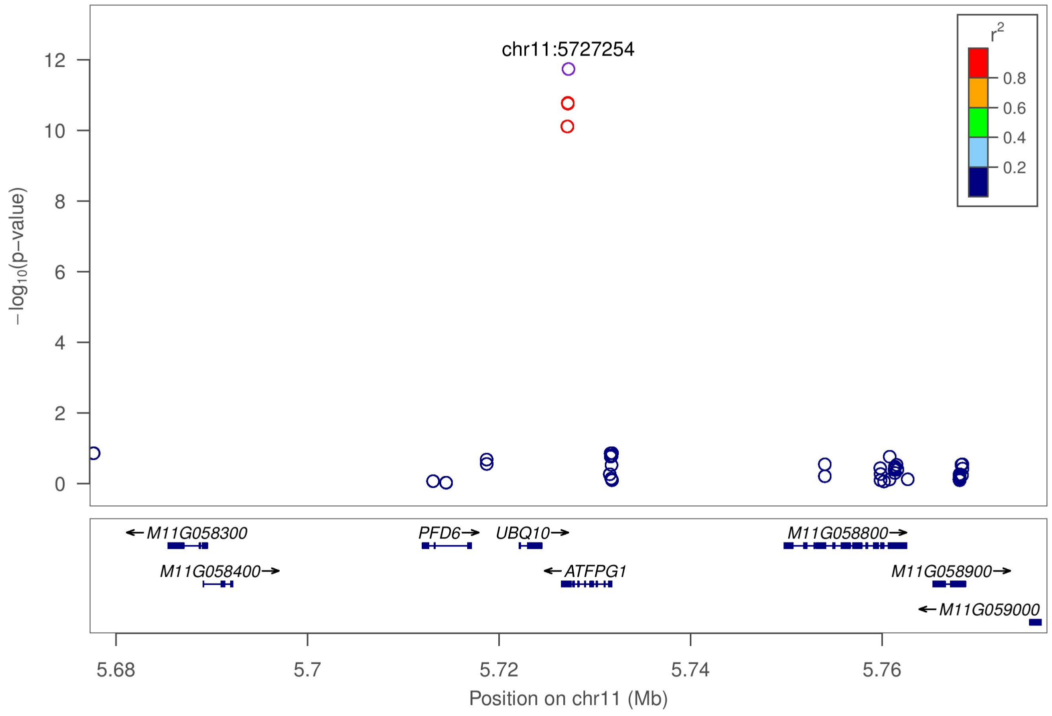

Apical pubescence (S12_5524524)

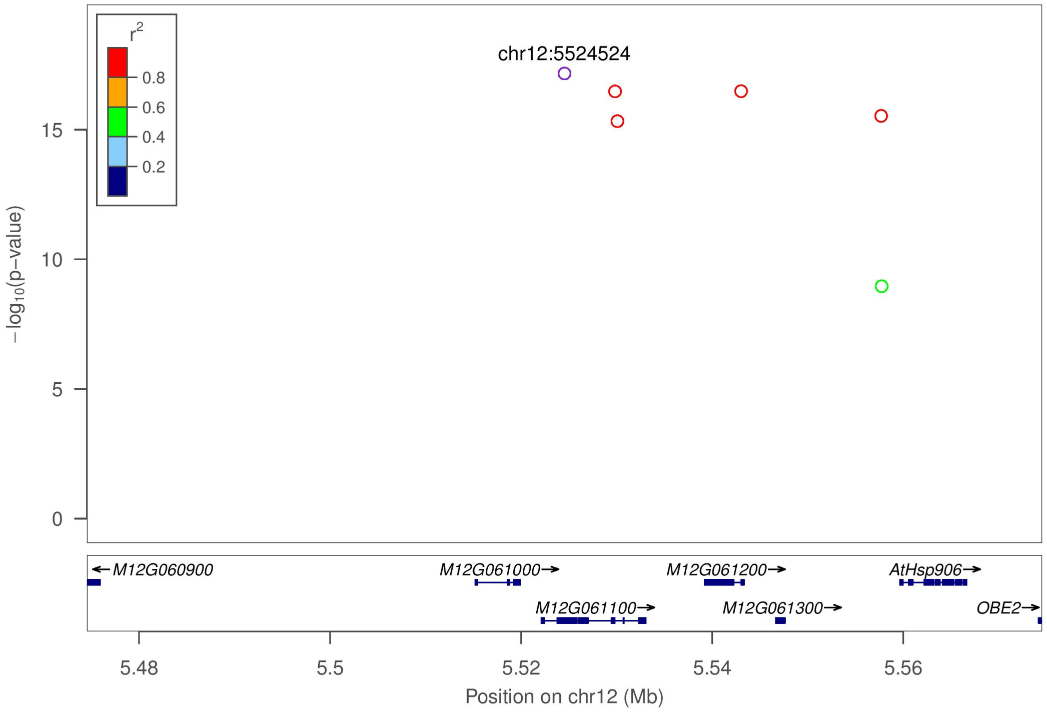

Apical pubescence (S16_1501762)

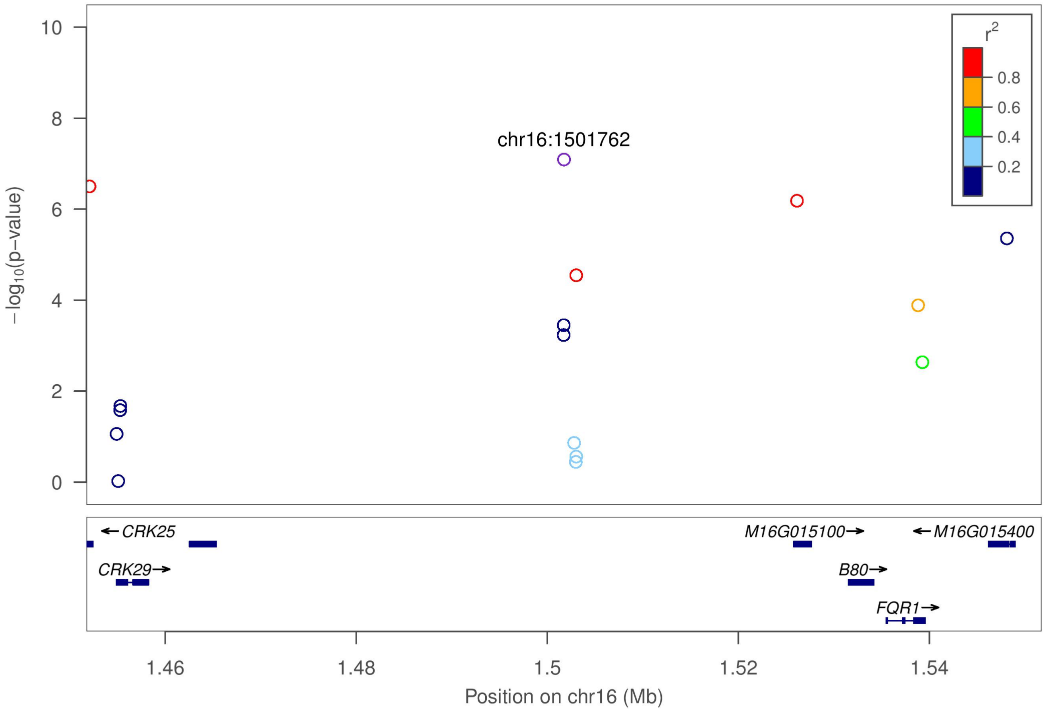

D

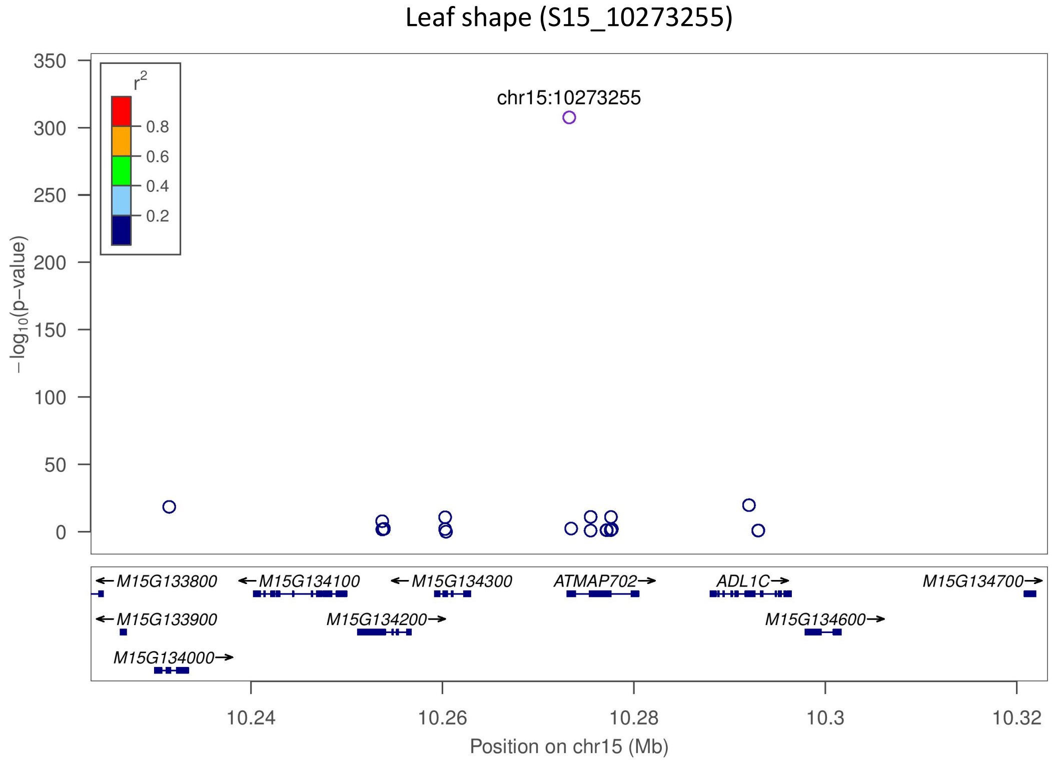
Leaf shape (S15_10273255)
r2
0.8
0.6
0.4
0.2
chr15:10273255
−log10(p−value)
350
300
250
200
150
100
50
0
M15G133800
M15G134100
M15G134300
ATMAP702
ADL1C
M15G134700
M15G133900
M15G134200
M15G134600
M15G134000
10.24
10.26
10.28
10.3
10.32
Position on chr15 (Mb)

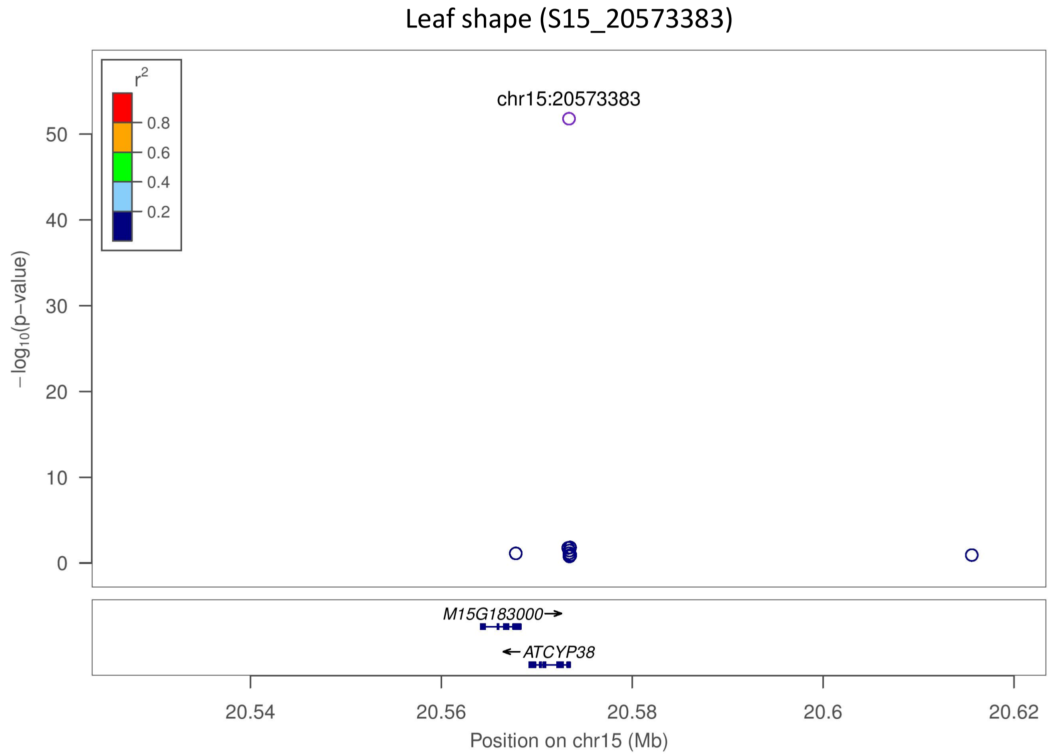
Leaf shape (S15_20573383)
r2
0.8
0.6
0.4
0.2
chr15:20573383
−log10(p−value)
50
40
30
20
10
0
M15G183000
ATCYP38
20.54
20.56
20.58
20.6
20.62
Position on chr15 (Mb)

E

Apical leaf colour (S2_6086714)

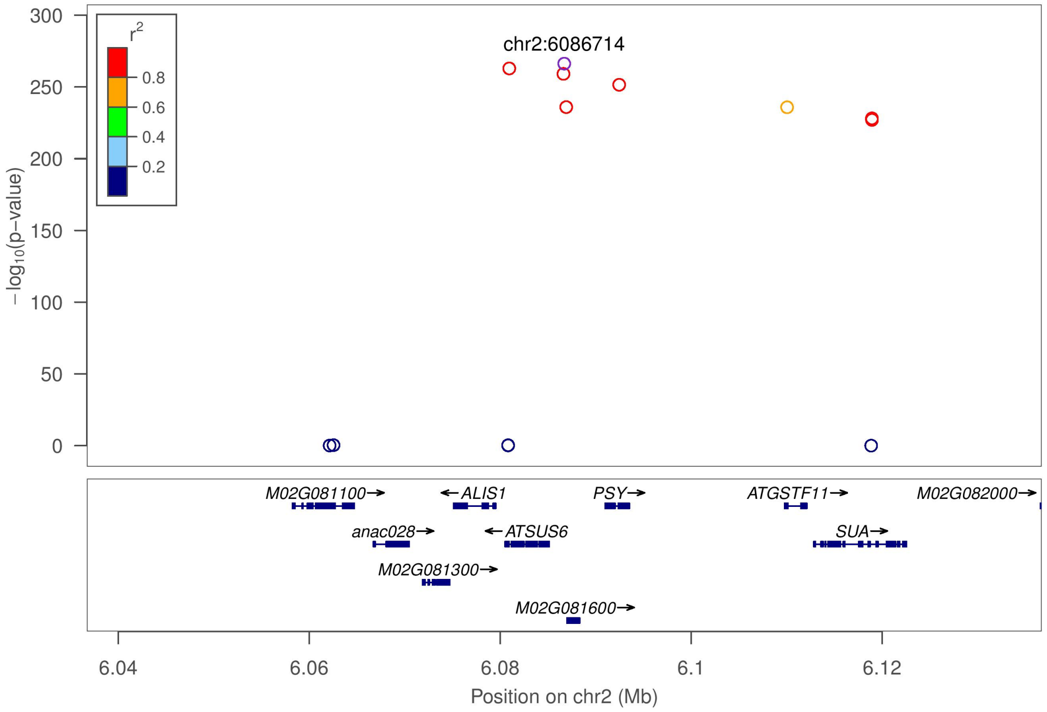

Apical leaf colour (S3_4745233)

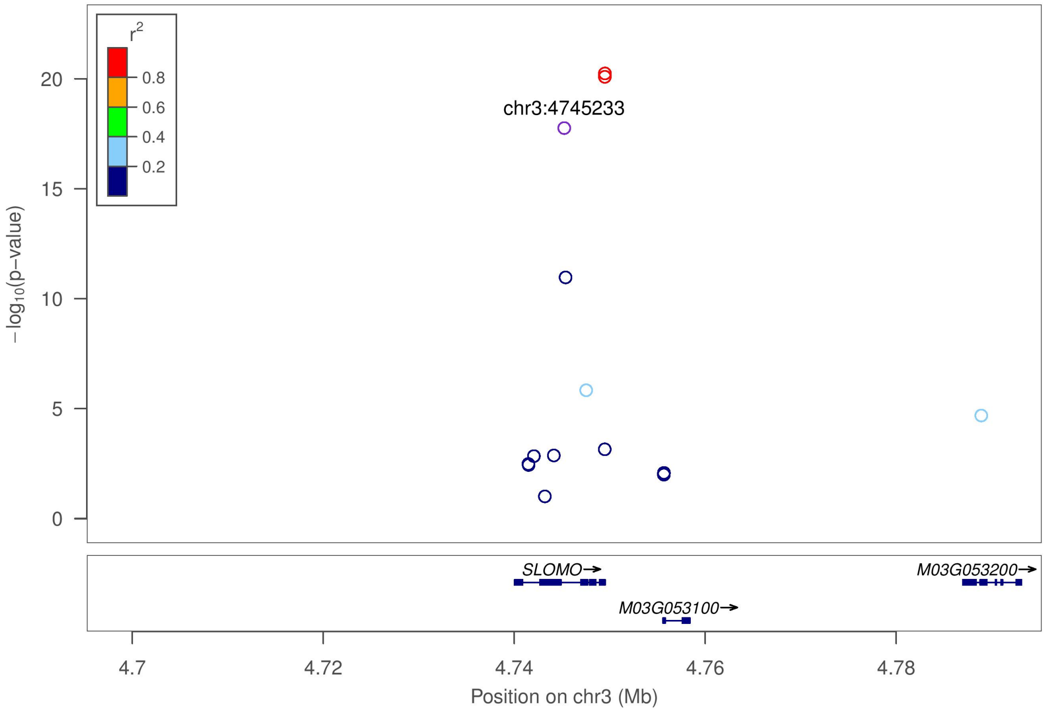

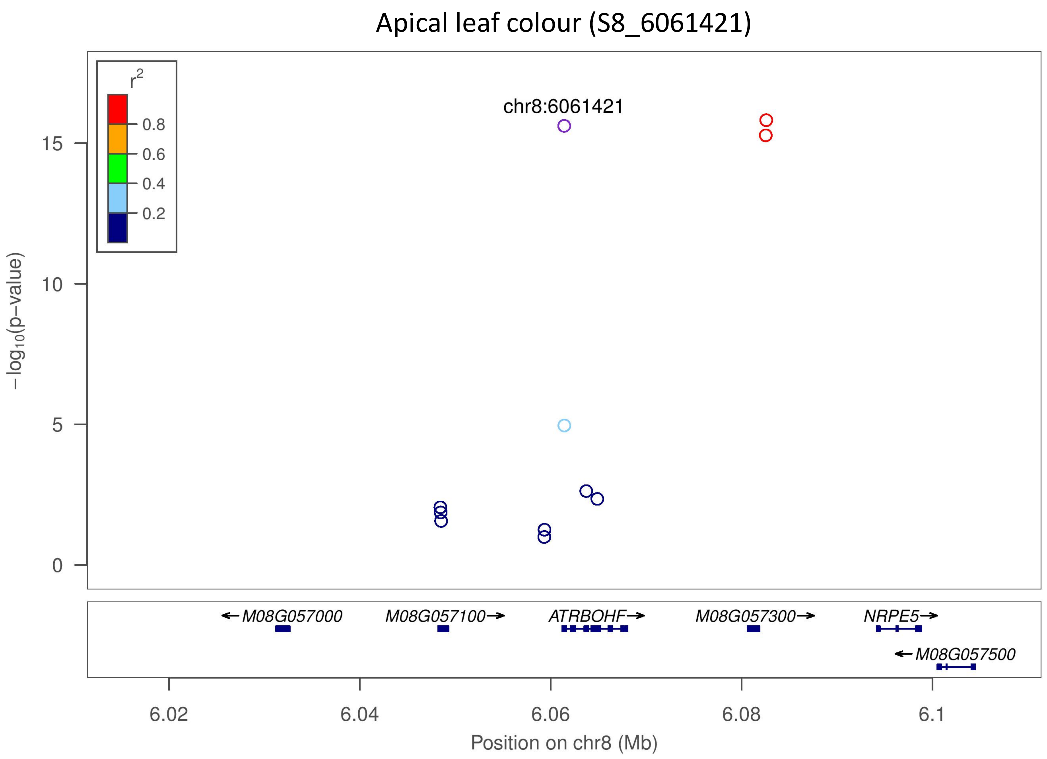

Apical leaf colour (S8_6061421)
r2
0.8
0.6
0.4
0.2
chr8:6061421
15
10
5
0
− log10(p−value)
M08G057000
M08G057100
ATRBOHF
M08G057300
NRPE5
M08G057500
6.02
6.04
6.06
6.08
6.1
Position on chr8 (Mb)

F

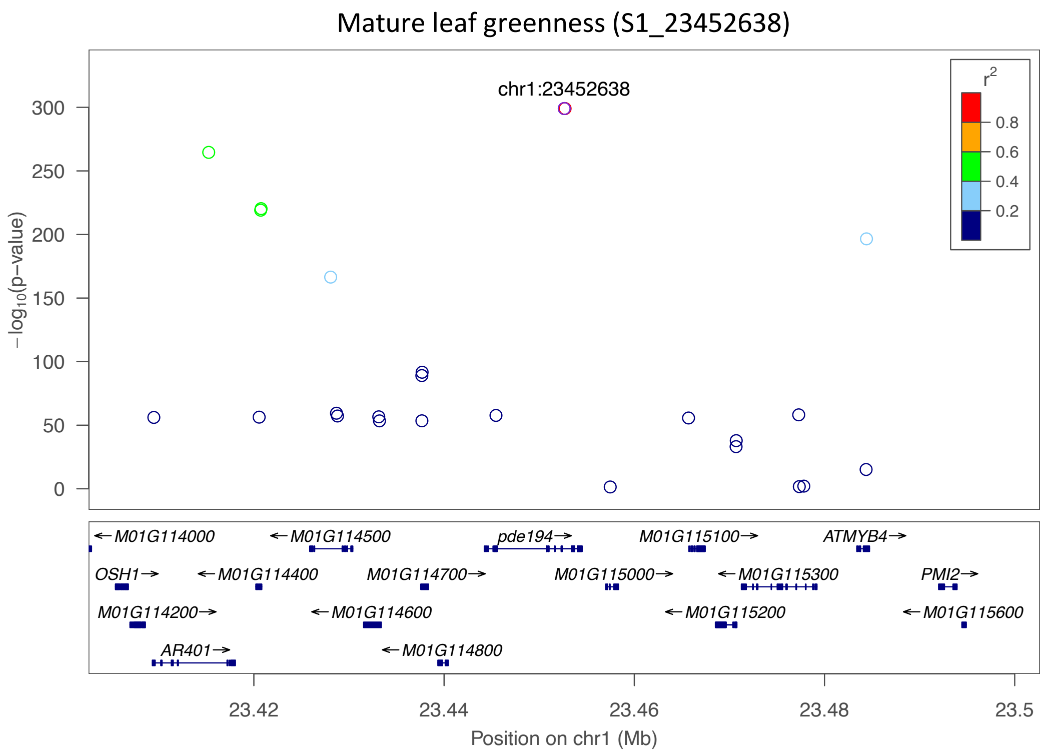

Mature leaf greenness (S1_23452638)
r2
0.8
0.6
0.4
0.2
chr1:23452638
300
250
200
150
100
50
0
− log10(p−value)
M01G114000
M01G114500
pde194
M01G115100
ATMYB4
OSH1
M01G114400
M01G114700
M01G115000
M01G115300
PMI2
M01G114200
M01G114600
M01G115200
M01G115600
AR401
M01G114800
23.42
23.44
23.46
23.48
23.5
Position on chr1 (Mb)

G

Petiole colour (S1_23055068)

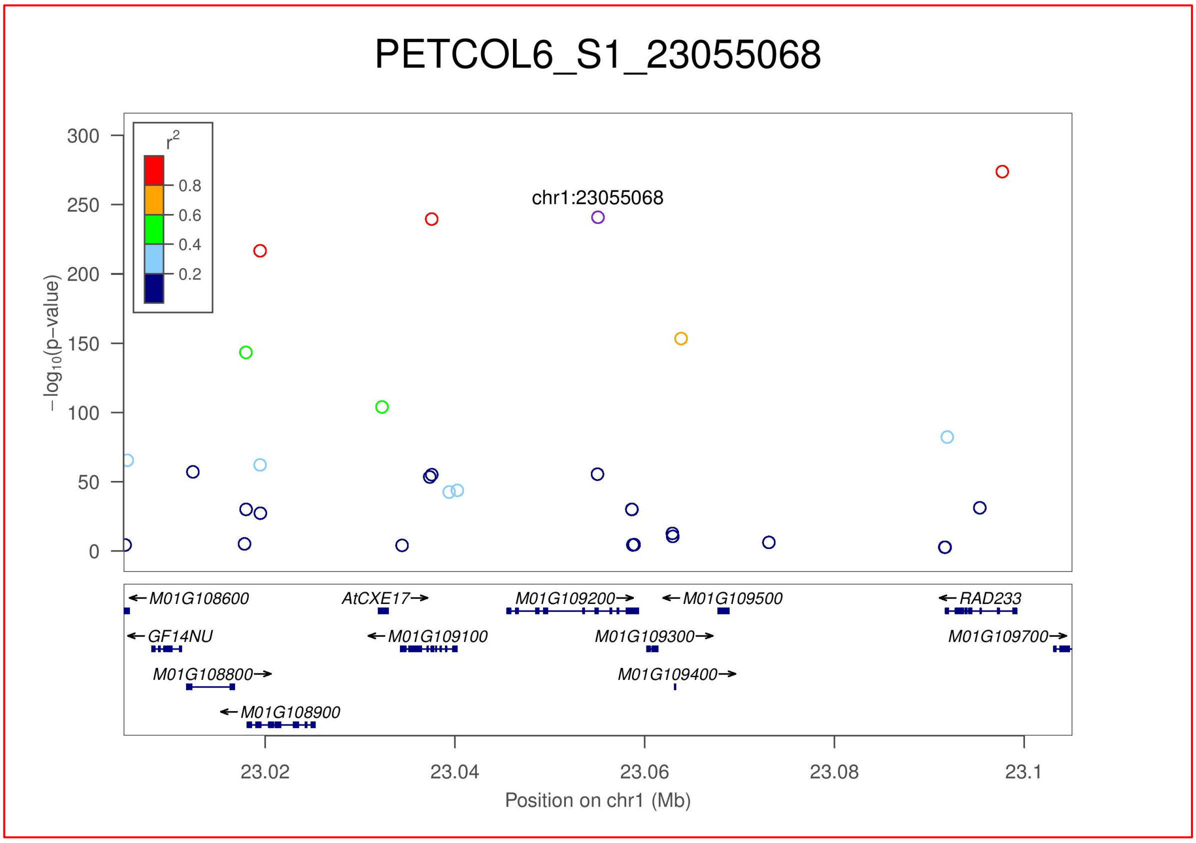

H

Harvest index (S2_2809137)

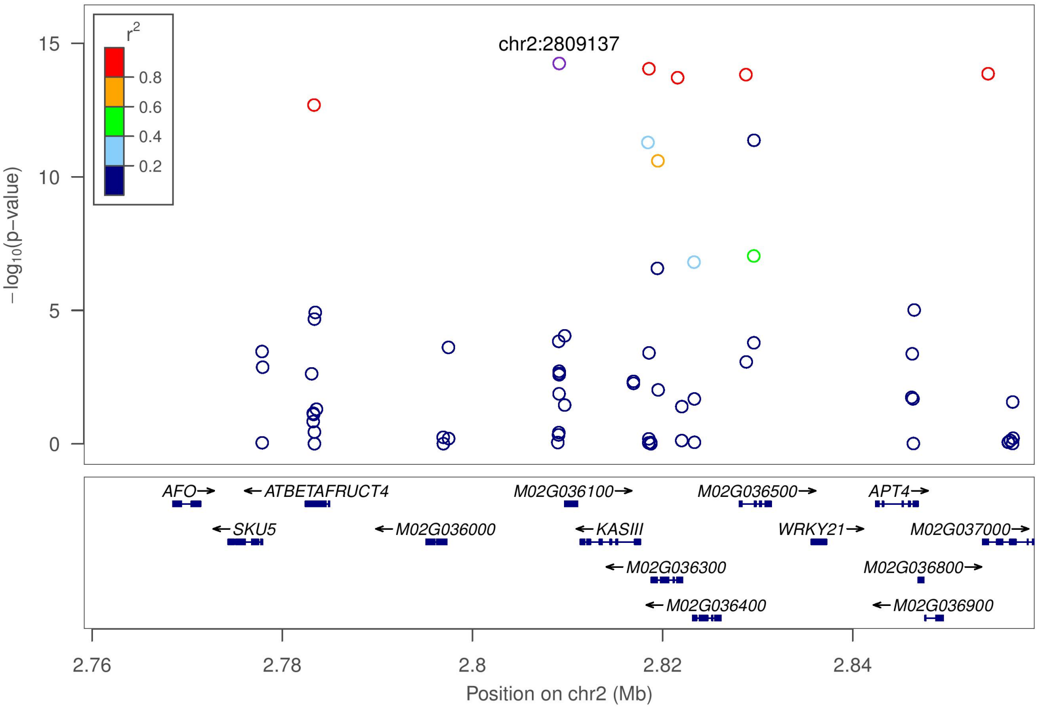

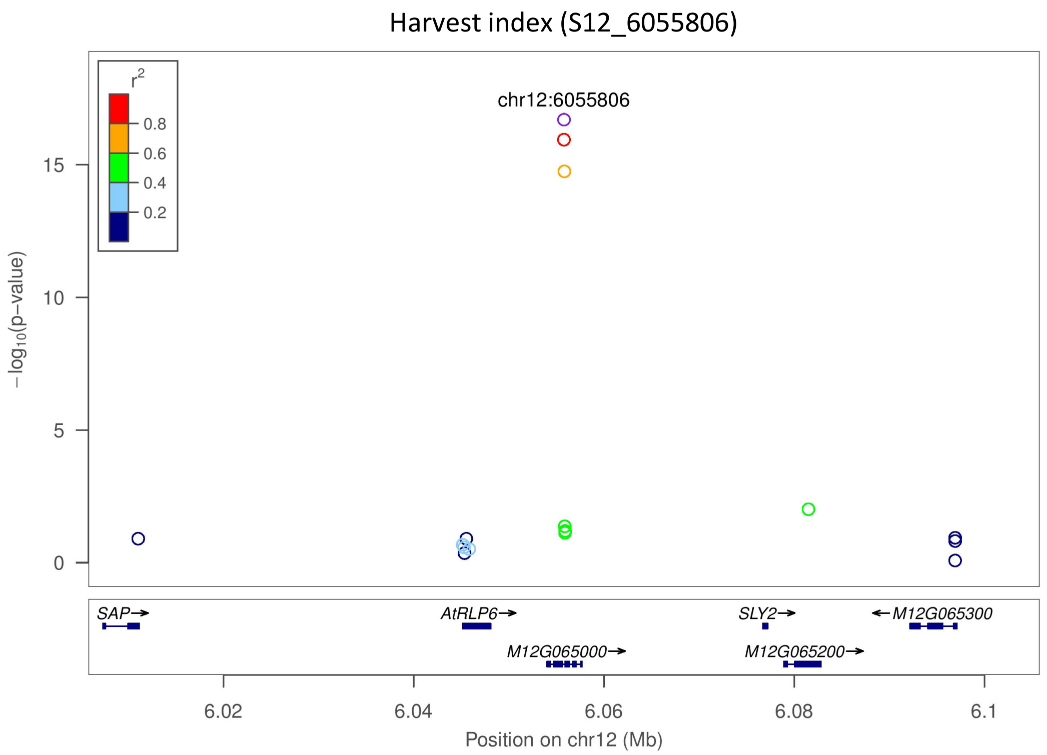
Harvest index (S12_6055806)
chr12:6055806
r2
0.8
0.6
0.4
0.2
−log10(p−value)
SAP
AtRLP6
M12G065000
SLY2
M12G065200
M12G065300
Position on chr12 (Mb)

I

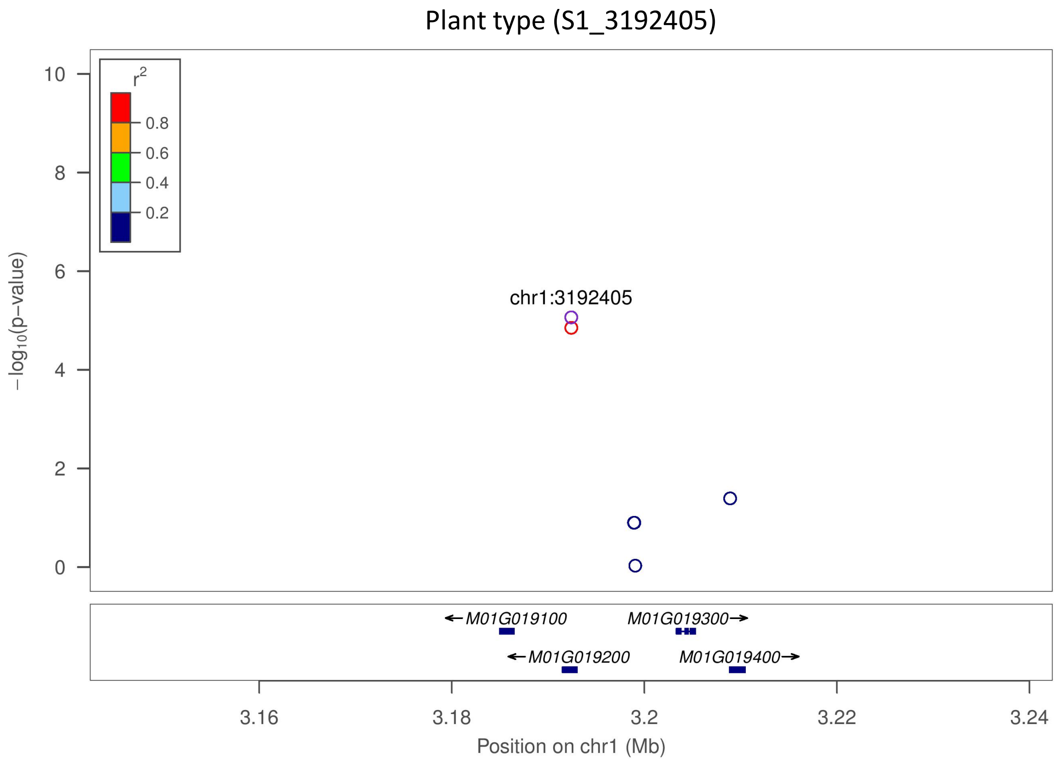
Plant type (S1_3192405)
chr1:3192405
r2
0.8
0.6
0.4
0.2
−log10(p−value)
M01G019100
M01G019200
M01G019300
M01G019400
Position on chr1 (Mb)

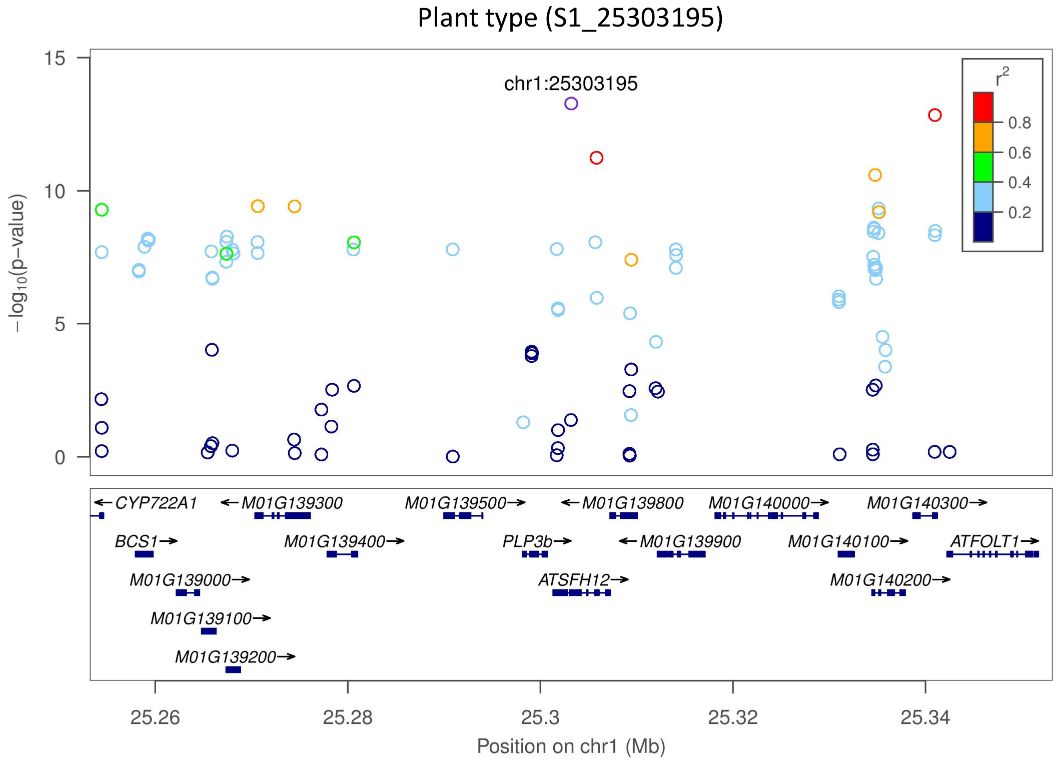
Plant type (S1_25303195)
chr1:25303195
r2
0.8
0.6
0.4
0.2
−log10(p−value)
15
10
5
0
CYP722A1
M01G139300
M01G139500
M01G139800
M01G140000
M01G140300
BCS1
M01G139400
PLP3b
M01G139900
M01G140100
ATFOLT1
M01G139000
ATSFH12
M01G140200
M01G139100
M01G139200
25.26
25.28
25.3
25.32
25.34
Position on chr1 (Mb)

J

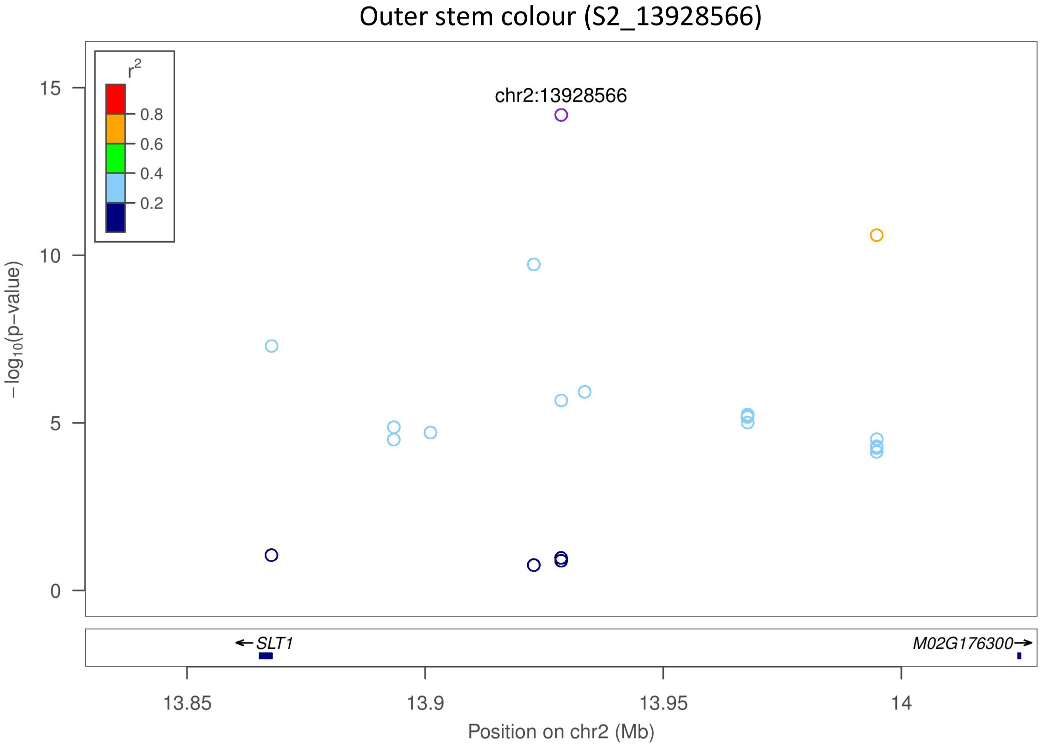
Outer stem colour (S2_13928566)
r2
0.8
0.6
0.4
0.2
chr2:13928566
−log10(p−value)
15
10
5
0
SLT1
M02G176300
13.85
13.9
13.95
14
Position on chr2 (Mb)

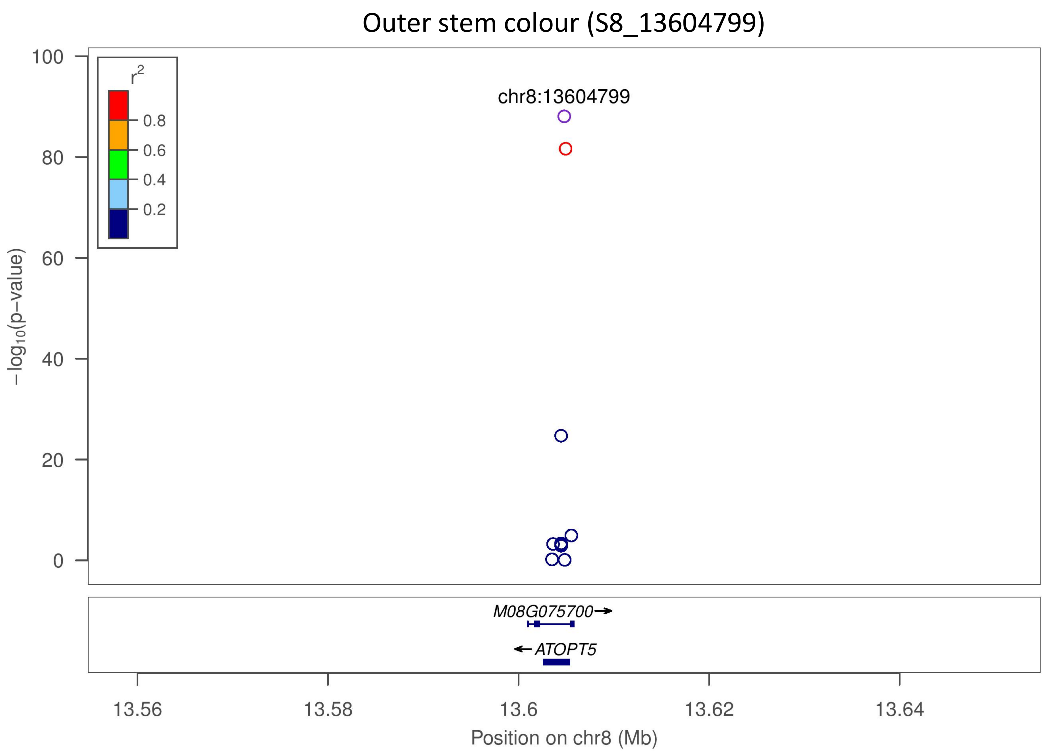
Outer stem colour (S8_13604799)
chr8:13604799
r2
0.8
0.6
0.4
0.2
−log10(p−value)
M08G075700
ATOPT5
Position on chr8 (Mb)

K

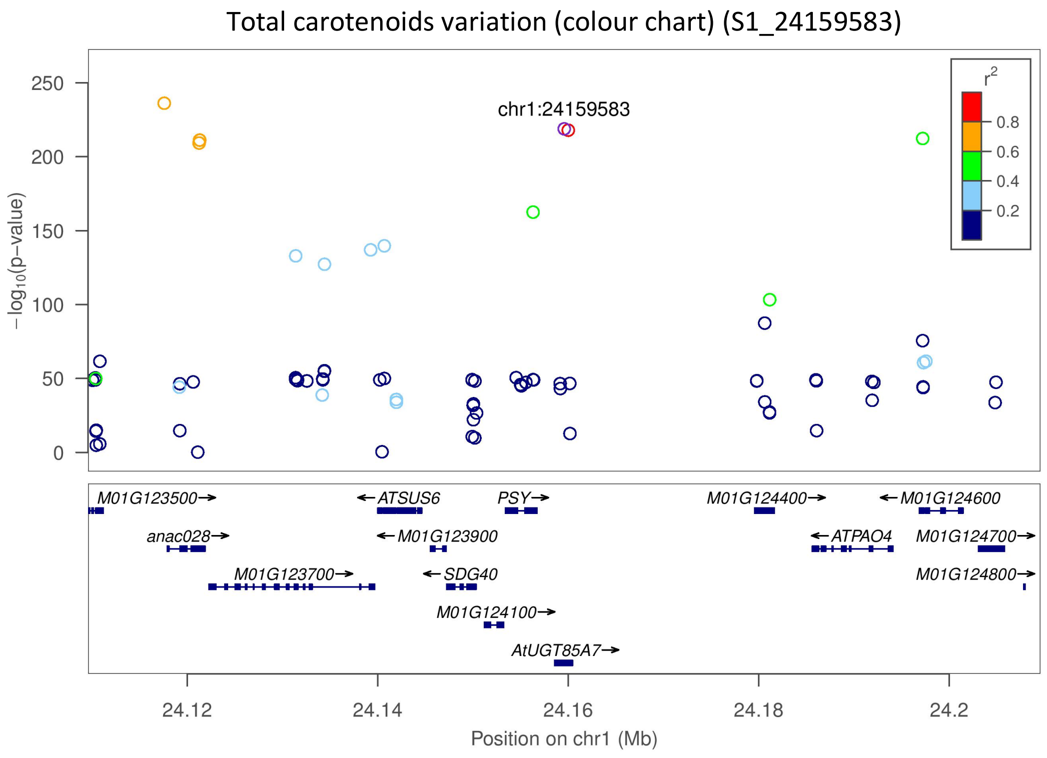
Total carotenoids variation (colour chart) (S1_24159583)
chr1:24159583
r2
−log10(p−value)
M01G123500
anac028
M01G123700
ATSUS6
M01G123900
SDG40
M01G124100
PSY
AtUGT85A7
M01G124400
ATPAO4
M01G124600
M01G124700
M01G124800
Position on chr1 (Mb)

Total carotenoids variation (colour chart) (S1_24636113)

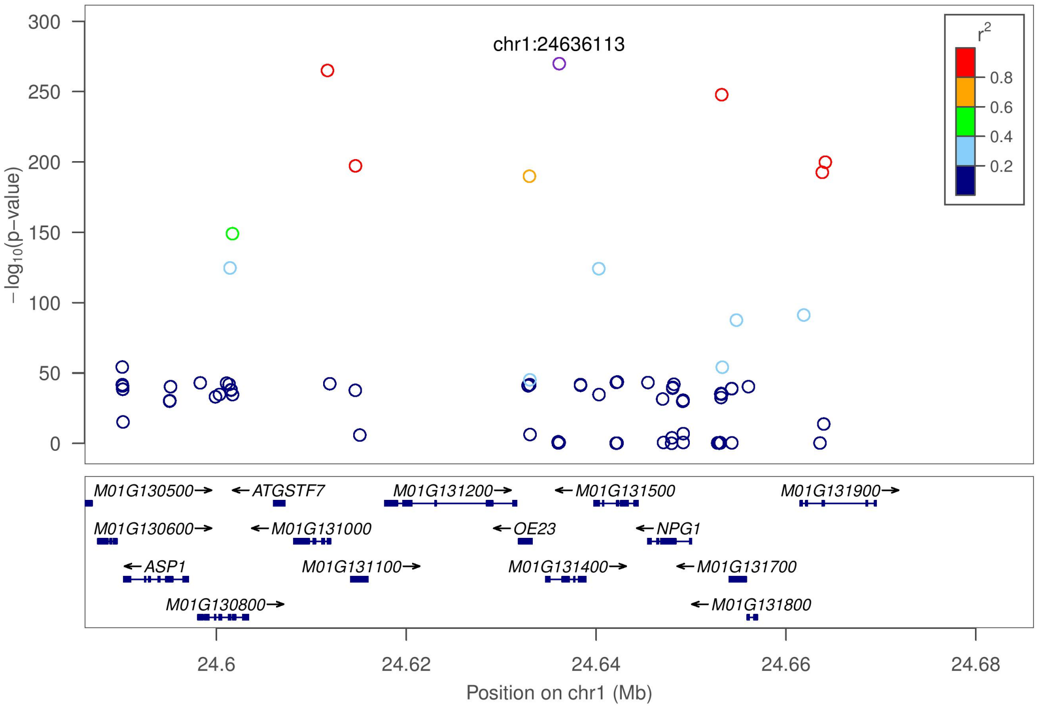

Total carotenoids variation (colour chart) (S1_30543962)

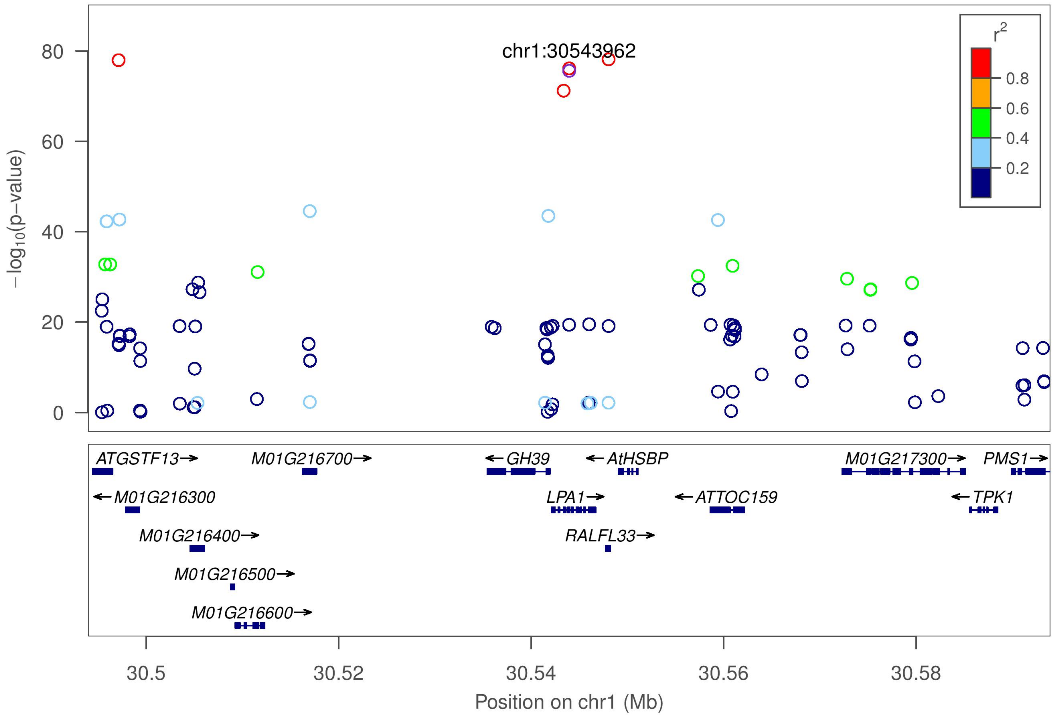

Total carotenoids variation (colour chart) (S5_3387558)

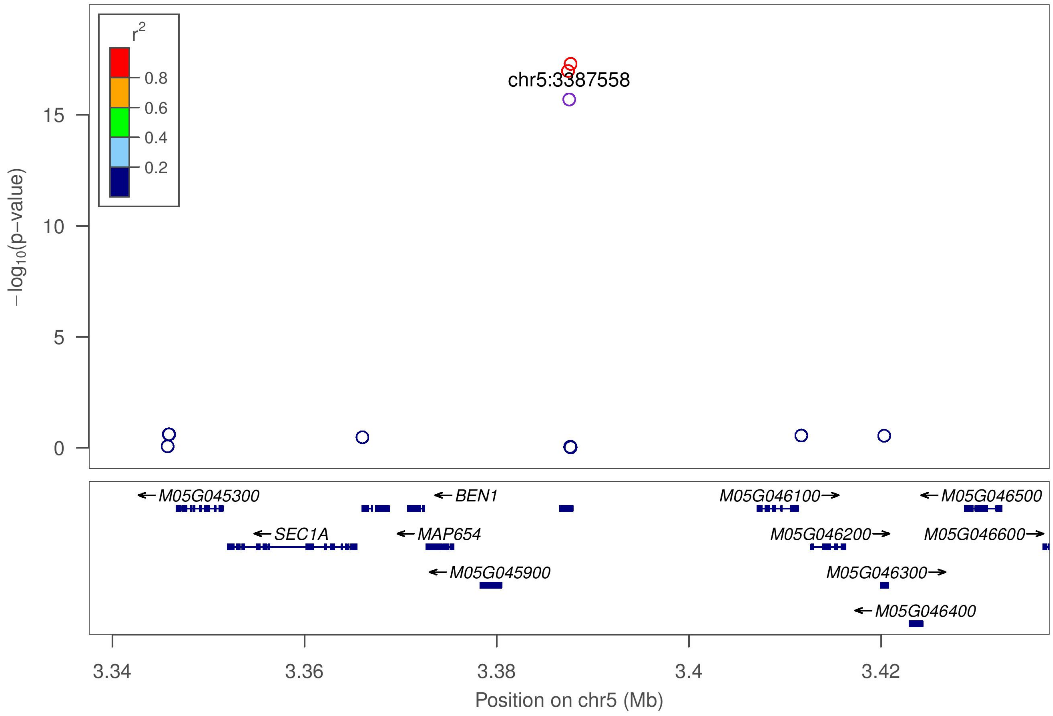

Total carotenoids (colour chart) (S8_4319215)

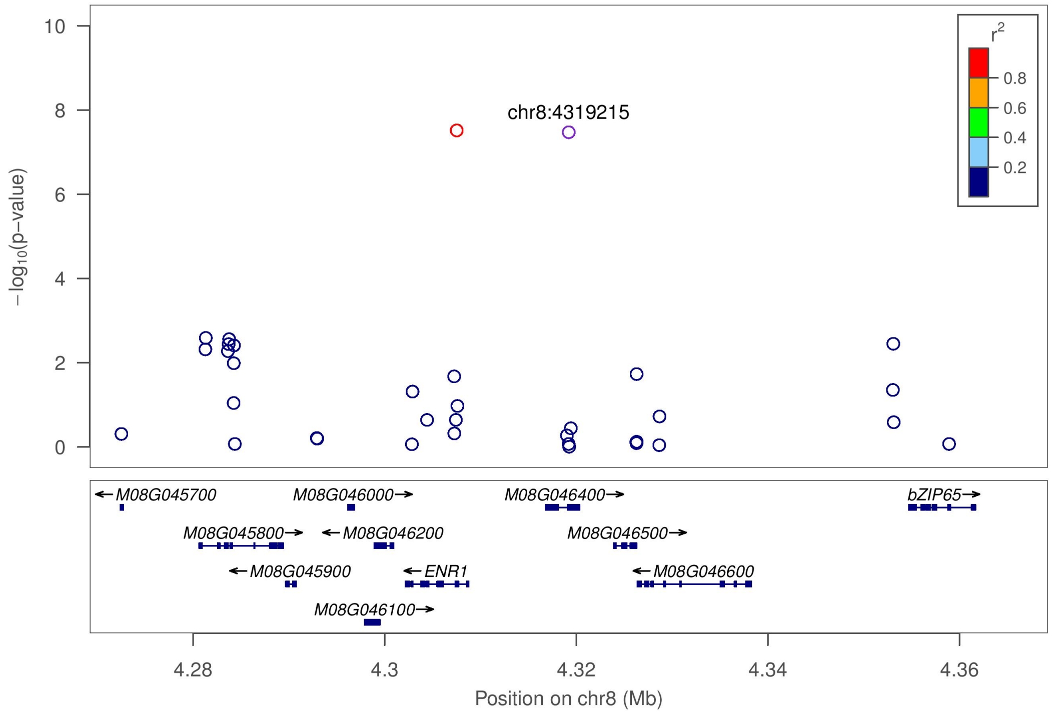

Total carotenoids variation (colour chart) (S8_25598183)

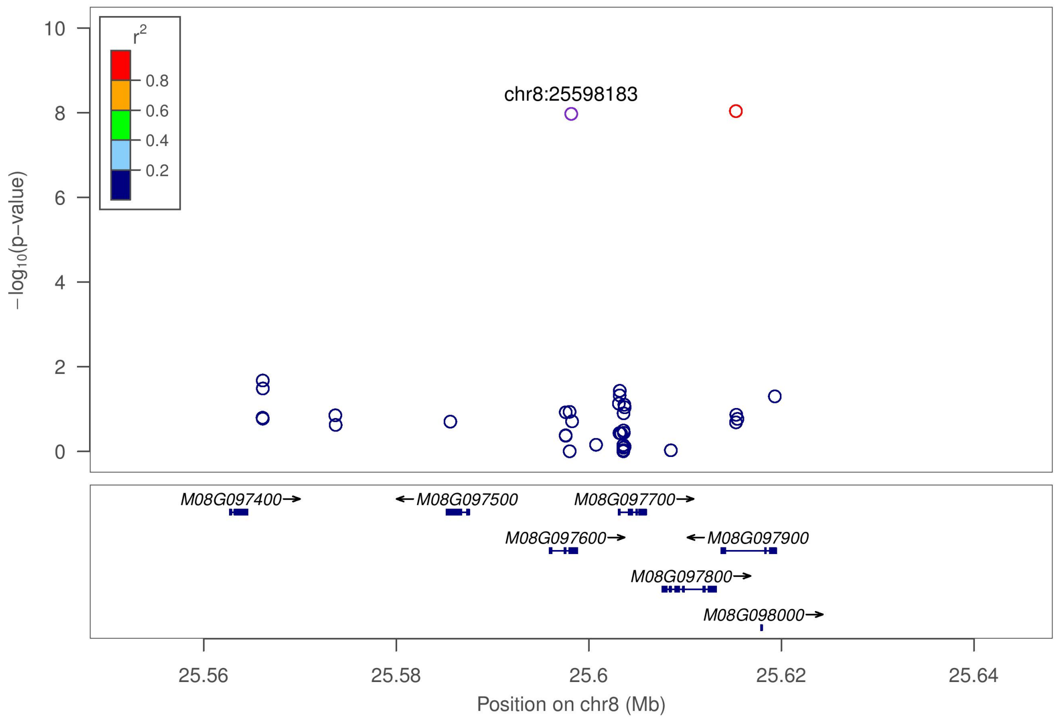

Total carotenoids variation (colour chart) (S15_7659426)

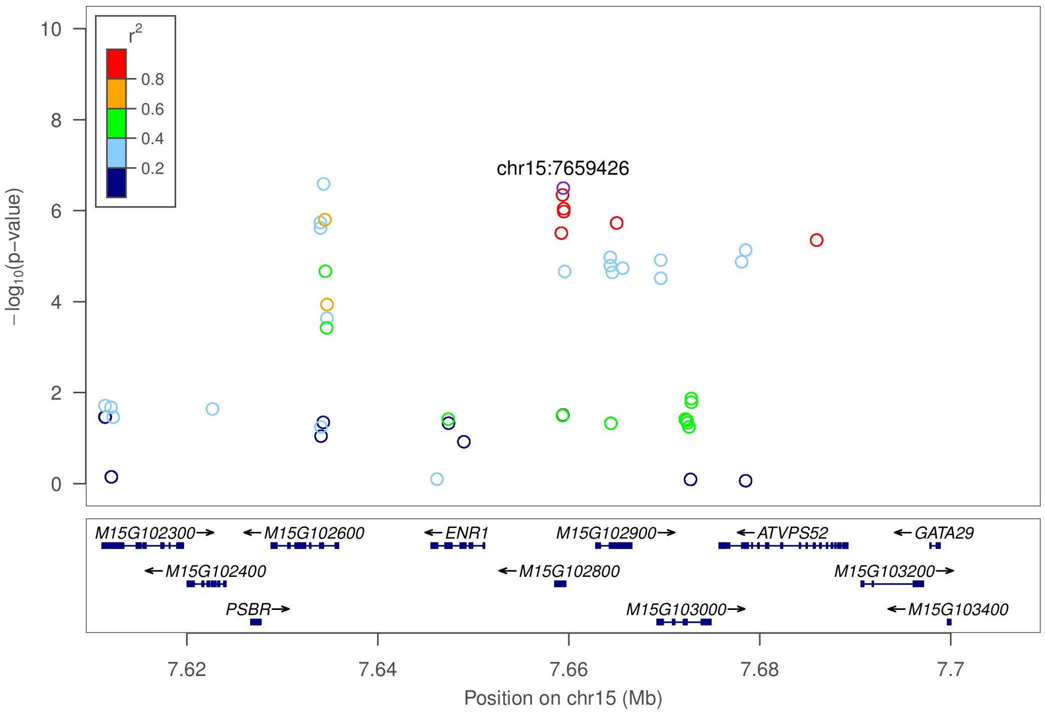

Total carotenoids variation (colour chart) (S16_484011)

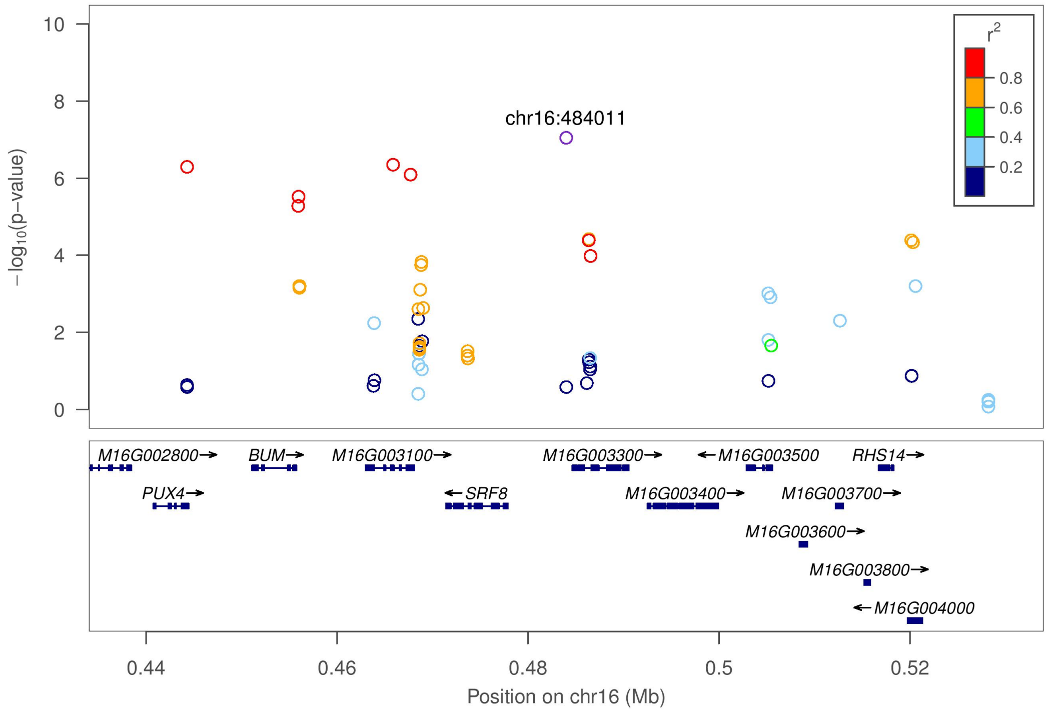

L

Dry matter content (S1_24636113)

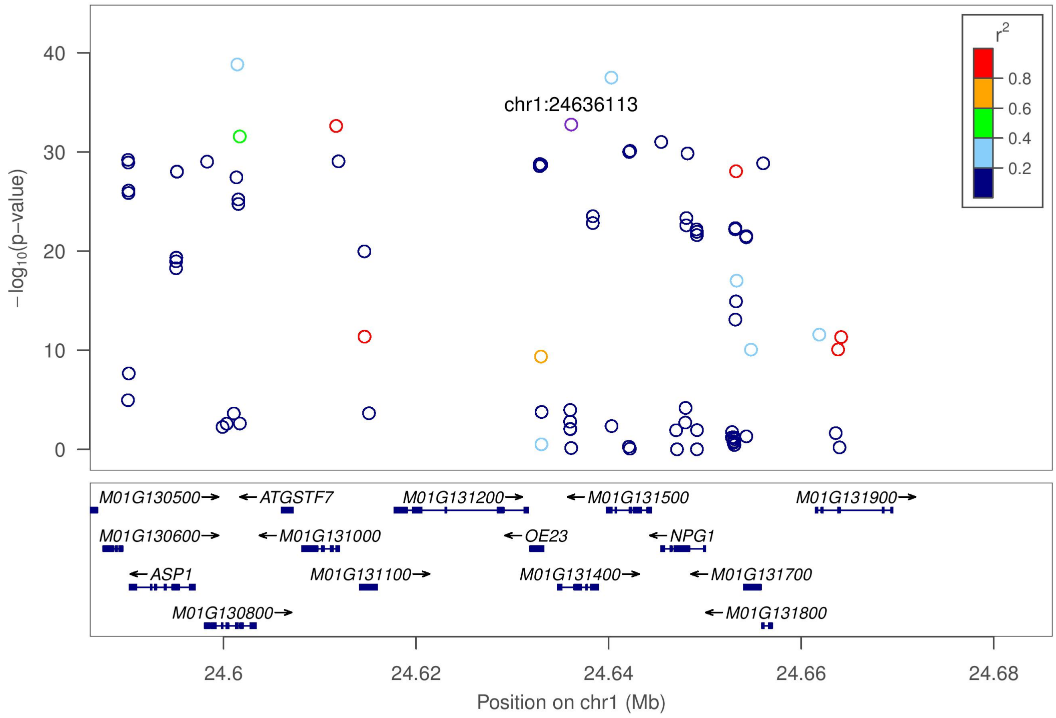

Dry matter content (S6_20589894)

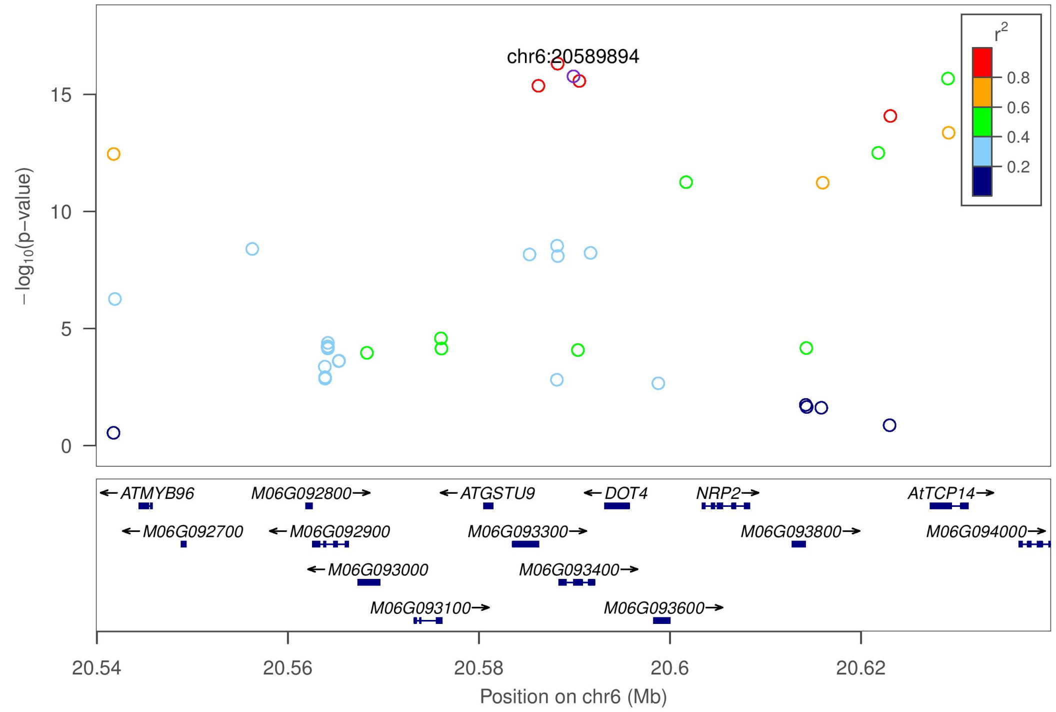

Dry matter content (S12_5524524)

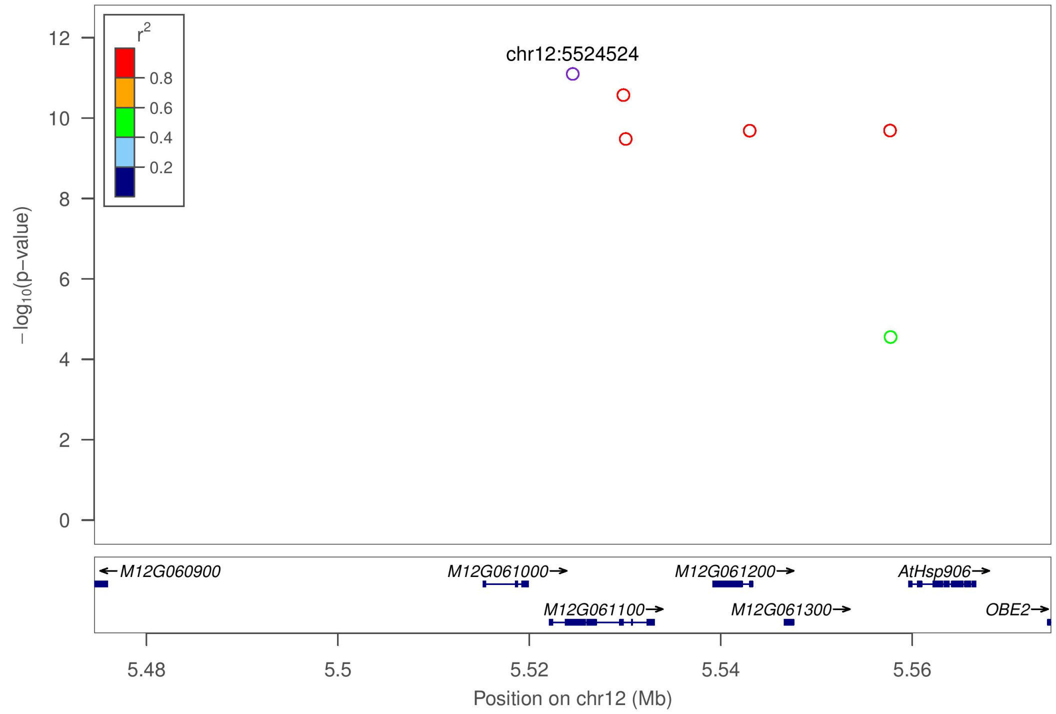

Dry matter content (S15_1012346)

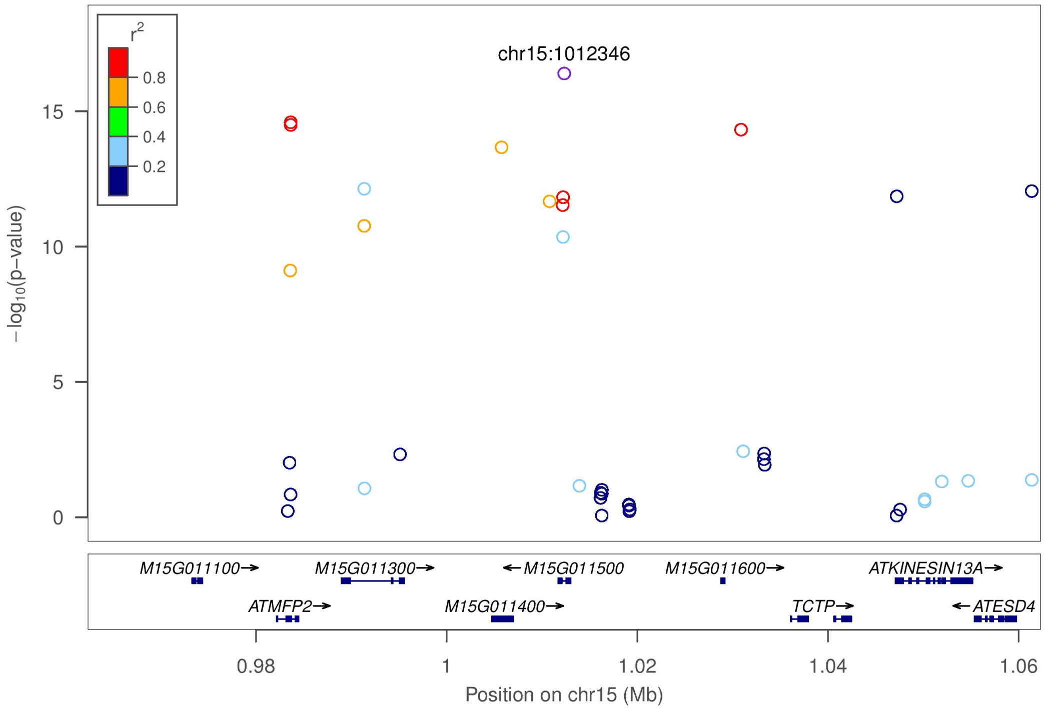

Dry matter content (S16_25663808)

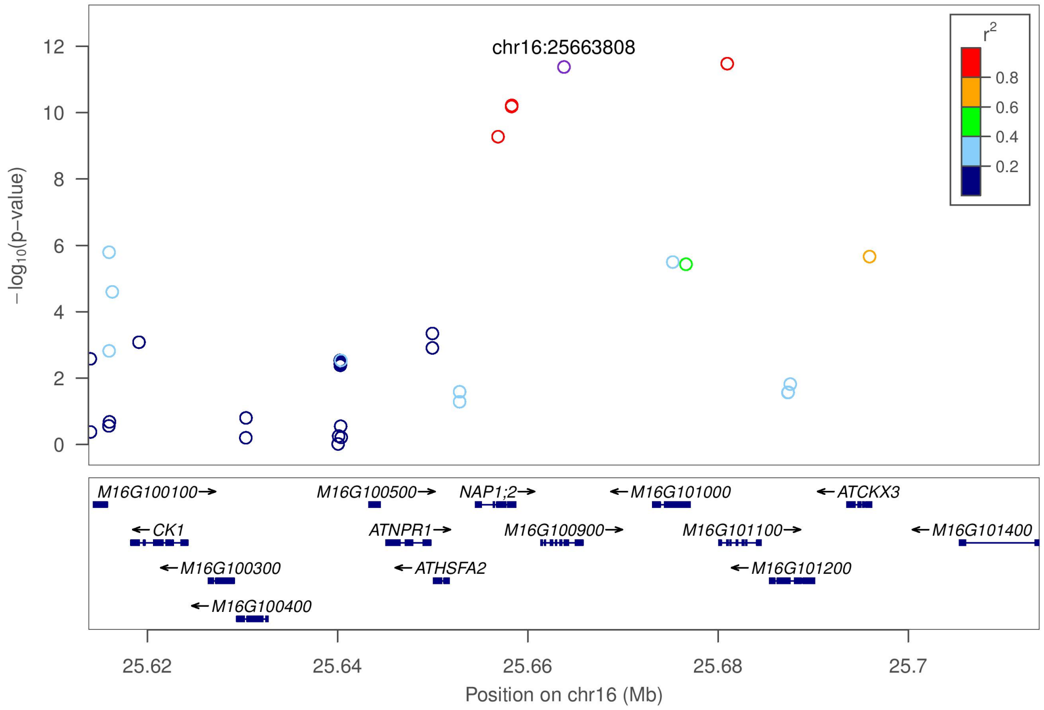

M

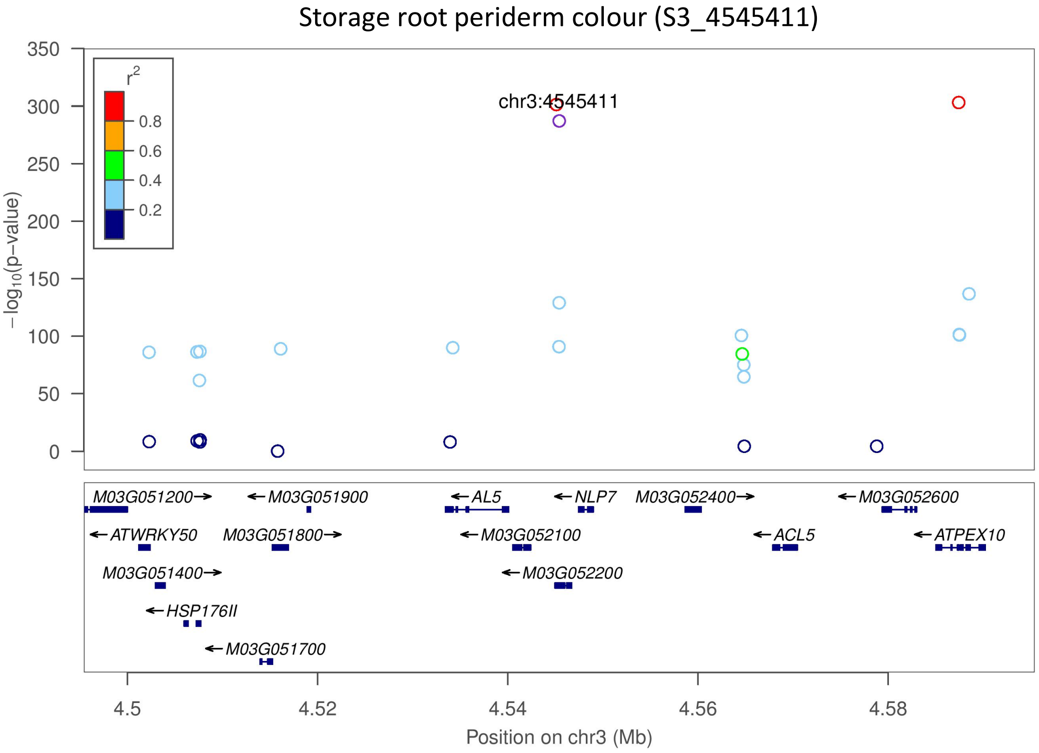
Storage root periderm colour (S3_4545411)
chr3:4545411
r2
0.8
0.6
0.4
0.2
−log10(p−value)
M03G051200
M03G051900
AL5
NLP7
M03G052400
M03G052600
ATWRKY50
M03G051800
M03G052100
ACL5
ATPEX10
M03G051400
M03G052200
HSP176II
M03G051700
4.5
4.52
4.54
4.56
4.58
Position on chr3 (Mb)

N

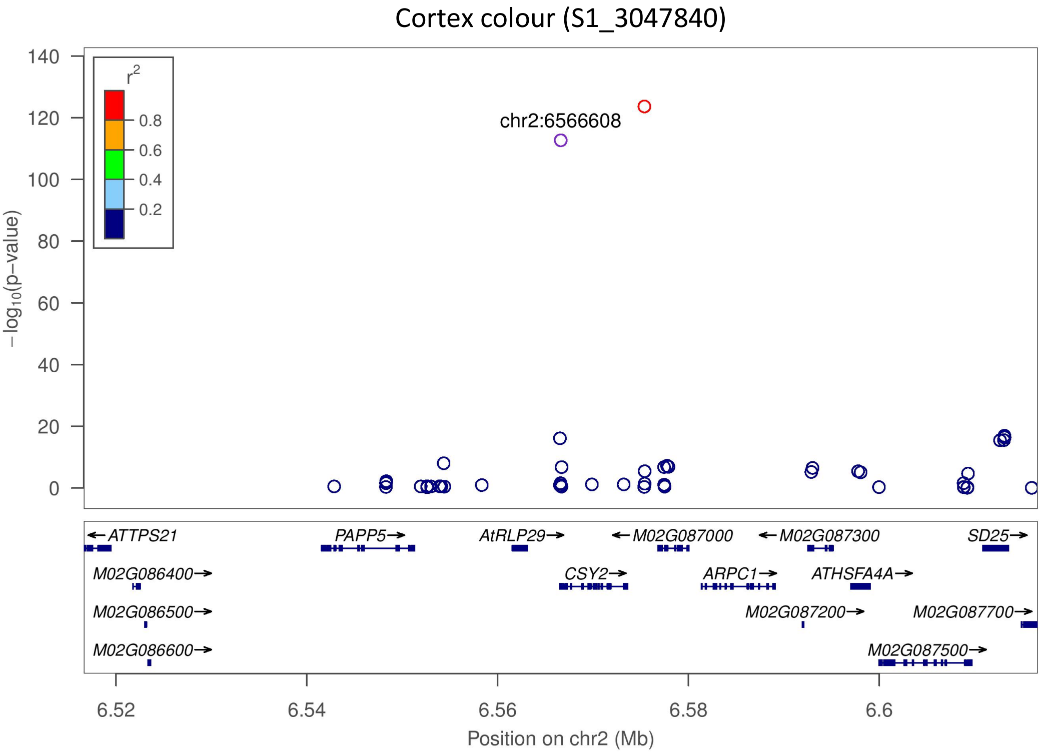
Cortex colour (S1_3047840)
chr2:6566608
r2
0.8
0.6
0.4
0.2
−log10(p−value)
ATTPS21
PAPP5
AtRLP29
M02G087000
M02G087300
SD25
M02G086400
CSY2
ARPC1
ATHSFA4A
M02G086500
M02G087200
M02G087700
M02G086600
M02G087500
6.52
6.54
6.56
6.58
6.6
Position on chr2 (Mb)

Cortex colour (S2_6566608)

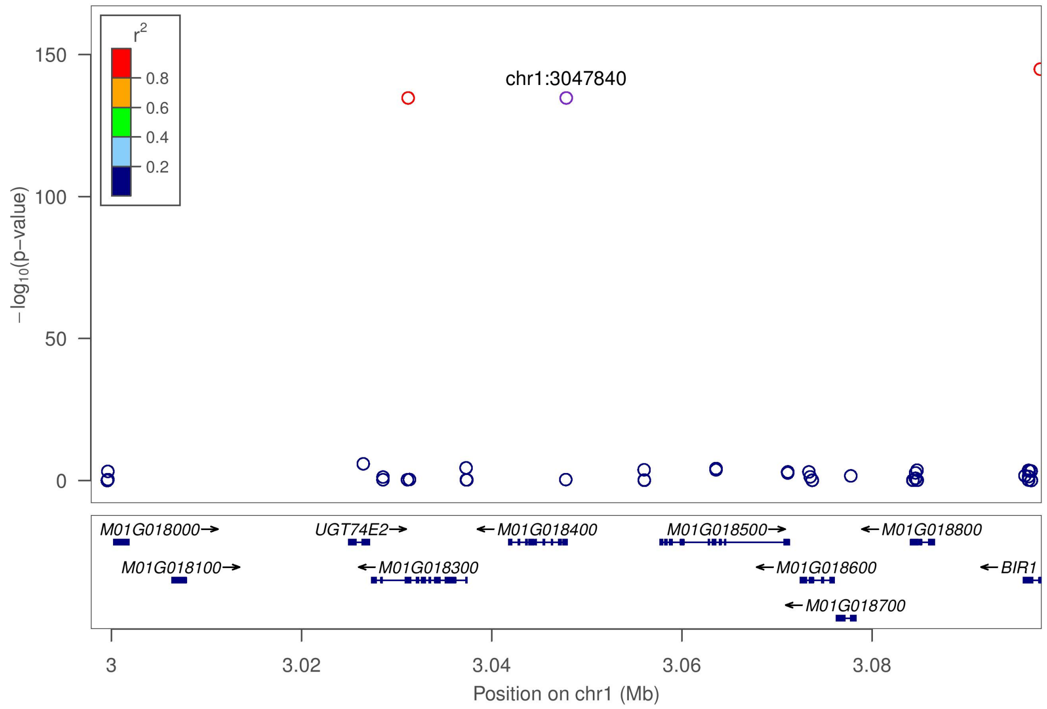

Figure S4 Regional Manhattan plots for each locus-trait combination. Plots include candidate genes within 100 Kb of the top SNP marker (50 kb upstream, 50 kb downstream) with some adjustments based on the extent of local linkage disequilibrium with the candidate marker. SNPs are color coded based on linkage disequilibrium with the top marker. (A) CMD severity; (B) CGM severity; (C) Apical leaf pubescence; (D) Leaf shape; (E) Apical leaf colour; (F) Mature leaf greenness; (G) Petiole colour; (H) Harvest index; (I) Plant type; (J) Outer stem colour; (K) Total carotenoids content; (L) Dry matter content; (M) Periderm colour; (N) Root cortex colour.
